# Effects of virtual reality interventions on anxiety symptoms in women undergoing gynecological examinations and surgeries: a multi-level dose–response meta-analysis

**DOI:** 10.3389/fpsyg.2026.1792559

**Published:** 2026-05-29

**Authors:** Yuehan Lv, Songwei Zhong, Tingxiao Zhang, Yan Zhang

**Affiliations:** 1 College of Physical Education, Liaoning Normal University, Liaoning, China; 2 Guangxi Science & Technology Normal University, Guangxi, China

**Keywords:** anxiety, gynecological examination, meta-analysis, surgery, systematic review, virtual reality

## Abstract

**Objective:**

Using a systematic review and multi-level meta-analysis, this study quantified the effects of virtual reality (VR) interventions on anxiety symptoms among women undergoing gynecological examinations or surgeries. Furthermore, it examined potential sources of effect heterogeneity and the relationship between intervention parameters and therapeutic efficacy, thereby providing an evidence-based reference for the rational application of VR interventions in clinical settings.

**Methods:**

This study followed the PRISMA guidelines. We conducted a systematic search of the PubMed, Web of Science, PsycINFO, and Cochrane Library databases up to January 5, 2026, and included randomized controlled trials (RCTs) that evaluated the effects of VR interventions on anxiety symptoms among women undergoing gynecological examinations or surgeries. A multi-level random-effects model was used to pool effect sizes, specifically the SMD. We also conducted heterogeneity assessments, publication bias tests, subgroup analyses, meta-regressions, and Bayesian dose–response analyses.

**Results:**

Twelve randomized controlled trials published between 2019 and 2025 were included. The meta-analysis showed that the VR intervention significantly reduced anxiety symptoms in women during the intervention period, with a small-to-moderate effect (SMD = −0.47, 95% CI − 0.73 to −0.21, *p* < 0.001). The quality of evidence was rated as moderate according to the GRADE criteria. Subgroup analyses suggested more consistent effects in non-invasive procedures, with VR alone, and when anxiety was assessed using the Numerical Rating Scale (NRS). Subgroup analyses suggested more consistent effects for interventions lasting 5–10 min, whereas no statistically significant effect was observed in highly invasive procedures. Linear meta-regression identified country/region, anxiety assessment tool, and methodological quality as significant moderators. Non-linear analyses suggested potential relationships with age, sample size, and intervention duration. A potential duration range of 9–13 min was identified, but this finding remained exploratory.

**Conclusion:**

Virtual reality interventions were associated with reduced anxiety symptoms, although substantial heterogeneity may limit the generalisability of the pooled effect. As a non-pharmacological digital intervention, VR may serve as a promising adjunct to routine care, particularly in less invasive gynecological settings. Further multicenter studies with larger samples and standardized designs are needed to verify long-term effects and clarify optimal intervention parameters.

**Systematic review registration:**

https://www.crd.york.ac.uk/PROSPERO/.

## Introduction

Anxiety is a future-oriented emotional state characterized by anticipation of potential negative events, with symptoms encompassing worry (verbal-subjective), avoidance (overt motor behavior), and muscle tension (somatic-visceral activity) (Craske et al., 2011). As one of the most prevalent mental health issues worldwide, anxiety disorders have increased by over 55% from 1990 to 2019 (Javaid et al., 2023). Women, in particular, face a heightened risk of developing anxiety disorders across the lifespan. This vulnerability is influenced by societal expectations surrounding gender roles, which often assign them greater responsibility for emotional management and caregiving. This chronic, often unrecognized psychological burden places them at an elevated risk of developing anxiety disorders throughout their lifespan (Wharton, 2009). Furthermore, women report higher levels of fear and more severe anxiety symptoms than men in both clinical and non-clinical contexts (Shear, 2008).

Gynecological examinations are fundamental components of women’s health management, as they play a crucial role in the early detection of gynecological diseases, risk assessment, and health maintenance. These procedures often involve exposure of personal privacy, manipulation of pelvic structures, and concerns about pain or abnormal results. Consequently, these procedures may induce tension, uneasiness, and fear in women (Hilden et al., 2003). Accordingly, research indicates that women experience varying levels of anxiety before gynecological examinations (Yilmaz and Demirel, 2021). This anxiety may lead some women to delay or avoid examinations, potentially affecting healthcare experiences and screening adherence (Oscarsson et al., 2008). The vast majority of women worldwide undergo at least one gynecological examination in their lifetimes, with many requiring multiple assessments (Yanikkerem et al., 2009).

Compared with gynecological examinations, anxiety related to surgery persists longer and may lead to more complex clinical implications. Consistent with this, research indicates that women experience elevated levels of anxiety during the pre-admission, perioperative, and postoperative phases (Johnston, 1980). This anxiety not only diminishes patients’ subjective healthcare experiences but also has the potential to interfere with medical procedures and prolong postoperative recovery (Tadesse et al., 2022). For instance, hysterectomy, recognized as the most common gynecological surgery in the United States, is frequently associated with infectious complications (Clarke-Pearson and Geller, 2013). From conception through Cesarean section (CS) and various surgeries involving female pelvic structures, including reproductive organs, women face numerous and complex physiological and medical challenges throughout their life course (Beckmann et al., 2013).

In this context, exploring a safe, non-pharmacological intervention for anxiety that does not add to the burden of medical procedures is crucial. VR can create immersive, controllable virtual environments, allowing individuals to engage with immersive multisensory experiences during medical procedures (Chirico et al., 2016). In one study, patients engaged their visual and auditory senses in a virtual environment by observing and listening to a virtual driver, which significantly reduced their anxiety (Dehghan et al., 2019). Mechanistically, previous research suggests that VR may reduce anxiety responses by engaging attentional resources and modulating emotional responses to medical stimuli (Gupta et al., 2018). In a randomized clinical trial, the VR intervention not only significantly reduced patient anxiety compared with the control group but also shortened operative time and improved staff satisfaction with the surgery (Wong and Choi, 2023). Compared with conventional psychotherapeutic approaches requiring longer intervention periods or additional personnel support, VR offers a practical, experience-based treatment approach that enables individualized, gradual, controlled, and immersive exposure, thereby minimizing patient distress and optimizing treatment success rates (Boeldt et al., 2019).

VR has shown potential benefits for anxiety reduction in women undergoing gynecological procedures (Alimonaki et al., 2025), such as hysteroscopy (Gamal et al., 2025) and Cesarean sections (Mohammadi et al., 2025). Relevant studies indicate that using VR during outpatient hysteroscopy significantly reduces patients’ anxiety (Cohen et al., 2024). Anxiety during colposcopy can be substantial, with approximately 80% of women experiencing varying degrees of tension and uneasiness during the examination. The VR intervention effectively reduced these anxiety responses during the procedure (Ceylan et al., 2025). Furthermore, research indicates that VR significantly reduces anxiety and psychological stress in women undergoing Cesarean sections. VR may improve relaxation and perioperative experience during Cesarean section procedures (Almedhesh et al., 2022). The intensity and duration of anxiety experienced by women may vary across procedural contexts, ranging from routine examinations with no invasive procedures to moderately invasive procedures that require instrument insertion, and further to highly invasive surgeries. Consequently, their responses to interventions are likely to differ based on the nature of the procedure. Although existing studies support the potential use of VR to manage anxiety in women undergoing gynecological examinations and surgeries, there are significant differences among these studies in procedural invasiveness, intervention implementation, intervention duration, anxiety assessment tools, and methodological quality. Consequently, this study conducted a systematic review and a multi-level meta-analysis to synthesize results from existing randomized controlled trials and identify potential moderators of the efficacy of VR interventions through subgroup analyses and both linear and non-linear meta-regressions. Bayesian dose–response analysis was additionally performed to explore the relationship between intervention duration and anxiety outcomes.

## Materials and methods

### Study design

This study was conducted as a systematic review of RCTs, following the guidelines of the Preferred Reporting Items for Systematic Reviews and Meta-Analyses (PRISMA) (Moher et al., 2015). Before screening the search results, the protocol was registered with the International Prospective Register of Systematic Reviews (PROSPERO) under registration number CRD420261277186, in accordance with the PRISMA statement.

### Study inclusion criteria

The inclusion criteria for this study were as follows: RCTs investigating the effects of VR interventions on anxiety symptoms in women undergoing gynecological examinations and surgeries. Studies were required to meet the following criteria simultaneously: (1) the intervention group implemented at least one structured VR intervention (including immersive or semi-immersive VR experiences) and compared its efficacy with passive control groups (no intervention), routine care, or standard medical/pharmacological interventions; (2) the study population consisted of women undergoing gynecological examinations or surgeries, including but not limited to hysteroscopy, colposcopy, gynecological surgery, and Cesarean sections, with no restrictions on race, region, or socioeconomic status; (3) the study employed validated, standardized anxiety assessment scales as primary outcome measures to evaluate changes in anxiety symptoms before and after the intervention; and (4) the literature was published in English and the full text was accessible. Exclusion criteria included non-experimental studies (e.g., theoretical studies, case reports), non-clinical studies (animal or cell experiments), secondary research (systematic reviews or meta-analyses), non-original research (duplicate publications, conference abstracts), gray literature (non-peer-reviewed research reports), and studies in which the intervention protocol lacked structure or standardization (e.g., single-session, non-standardized psychological comfort or counseling interventions). Although systematic reviews did not meet the final inclusion criteria, their reference lists were manually screened as potential sources to minimize the omission of relevant studies.

### Search strategy

This study systematically searched the PubMed, Web of Science, PsycINFO, and Cochrane Library databases through January 5, 2026, to comprehensively identify RCTs examining the effects of VR interventions on anxiety symptoms in women undergoing gynecological examinations and surgeries. The search strategy was developed using the PICOS framework, focusing on female populations undergoing gynecological examinations or surgeries (P), with VR intervention (≥1 session) as the intervention (I), and no treatment, routine care, or traditional medical/pharmacological therapy as the control (C). The primary outcome (O) was the change in standardized anxiety symptom scores, and the study design was limited to RCTs (S). During the search process, subject headings were combined with free-text terms. Core search terms included, but were not limited to: (‘Virtual Reality’ OR ‘VR’ OR ‘Virtual Reality Exposure Therapy’ OR ‘VRET’) AND (‘Women’ OR ‘Female’ OR ‘Woman’ OR ‘Women’s Health’ OR ‘Gynecologic Examination’ OR ‘Gynecologic Surgery’ OR ‘Hysteroscopy’ OR ‘Colposcopy’ OR ‘Cesarean Section’) AND (‘Anxiety’ OR ‘Anxiety Symptoms’ OR ‘Anxiety Disorders’) AND (‘Randomized Controlled Trial’ OR ‘RCT’). The search strings were adjusted to match the specific retrieval rules of each database; the complete search strategies for each database are detailed in the Supplementary document: Retrieval Strategy.

### Study selection process

The literature screening process followed the PRISMA guidelines. Initially, all literature from the systematic search was imported into the reference management software Zotero 7.0 for de-duplication. Subsequently, two researchers independently screened titles and abstracts to exclude studies that clearly did not meet the inclusion criteria. Full texts were retrieved for studies that passed the initial screening, and their eligibility regarding study design, participants, interventions, control conditions, and outcome measures was comprehensively evaluated using the PICOS framework. Any discrepancies during screening were resolved through discussion and adjudication by a third researcher to minimize potential selection bias. During the data extraction phase, a standardized data extraction form was used. Two researchers independently entered data in parallel and cross-verified key variables (such as sample characteristics and intervention implementation parameters). Any disagreements were resolved through discussion within the research team to reach a final consensus.

### Data synthesis

This study conducted all meta-analyses in the R statistical environment (version 4.3.1), primarily using the meta, metafor, diamet, and ggplot2 packages. For continuous outcomes, because individual studies may report multiple outcome measures or results at various time points, the primary analysis was conducted within a multilevel meta-analysis framework to mitigate bias arising from potential correlations among effect sizes. To ensure comparability across measurement tools, the Mean Difference (MD) or Standardized Mean Difference (SMD) was consistently used as the effect size metric. Specifically, the SMD was calculated using Hedges’ g to correct for small-sample bias, and effect size magnitude was interpreted using established thresholds: g = 0.2 indicated a small effect, 0.5 a medium effect, and ≥ 0.8 a large effect (Nakagawa and Cuthill, 2007). Pooled effect sizes were calculated using the inverse-variance weighting method. The primary analysis used a random-effects model with the restricted maximum-likelihood (REML) estimator, and results from a fixed-effect model were also reported when heterogeneity was low (*I*^2^ < 50%). Between-study heterogeneity was assessed using Cochran’s *Q* test, with a *p*-value less than 0.10 indicating significant heterogeneity, and the I^2^ statistic, with values exceeding 50% indicating high heterogeneity (Lau et al., 1997). Publication bias was assessed using a combination of visual inspection of funnel plots and Egger’s linear regression test. The trim-and-fill method was used to adjust the pooled effect estimates when funnel plot asymmetry was detected (Maier et al., 2022). Potential high-influence studies were identified using standardized residuals (|*Z*| > 2.5) and Cook’s distance exceeding three times the mean (Viechtbauer and Cheung, 2010). To examine the robustness of the results, several sensitivity analyses were conducted: (1) the leave-one-out method, using the metainf function, was employed to assess the influence of individual studies on the overall effect; (2) meta-regression analyses based on the REML method were performed to explore the impact of potential moderators, such as intervention duration and sample characteristics, on effect size, with regression trends visualized using bubble plots (Van Houwelingen et al., 2002); (3) subgroup analyses were conducted to investigate potential sources of heterogeneity. To ensure the comparability of results across different measurement tools, the Hedges–Olkin method was used to convert the raw data as follows $\mathrm{SMD}=\frac{M_{\text{Intervention}}-M_{\text{Control}}}{{\mathrm{SD}}_{\text{Pooled}}},{\mathrm{SD}}_{\text{Pooled}}=\sqrt{\frac{(n_{1}-1){\mathrm{SD}}_{1}^{2}-(n_{2}-1){\mathrm{SD}}_{2}^{2}}{n_{1}+n_{2}-2}}$ (Hedges and Olkin, 2014). To assess the reliability of cumulative evidence, Trial Sequential Analysis (TSA) was introduced to estimate the required information size (RIS/DIS). The RIS/DIS, along with the current cumulative sample size and cumulative *Z*-values, is reported together to determine whether the existing evidence meets the criteria for sufficiency (Wetterslev et al., 2008). In addition, a cumulative meta-analysis was conducted in publication order. The cumulative SMD and its 95% confidence intervals were updated dynamically to identify the time point at which a statistically significant effect first emerged and to examine the stability of the evolution of effect sizes over time (Lau et al., 1995). To further enhance estimation precision under conditions of sparse exposure data and small sample sizes, a Bayesian hierarchical dose–response model was developed. Restricted cubic splines were used to model non-linear relationships, and parameter estimation was performed by combining weakly informative priors with Markov Chain Monte Carlo (MCMC) methods (Crippa and Orsini, 2016; Hamza et al., 2021).

### Risk of bias (quality) assessment

This study used the most recent version of the Risk of Bias tool for randomized trials (RoB 2, 2019 update), as released by the Cochrane Collaboration, to systematically assess the risk of bias across all included studies. The evaluation encompassed several domains: bias arising from the randomization process, bias due to deviations from intended interventions, bias resulting from missing outcome data, bias in the measurement of outcomes, and bias in the selection of reported results (Sterne et al., 2019). The assessment was performed independently by two researchers, and judgments were categorized into three levels: “low risk,” “some concerns,” and “high risk.” Any discrepancies were resolved through review and discussion with a third researcher, and a final consensus was reached to maximize the evaluation process’s objectivity, consistency, and reliability.

## Results

### Study selection

**Figure 1:**
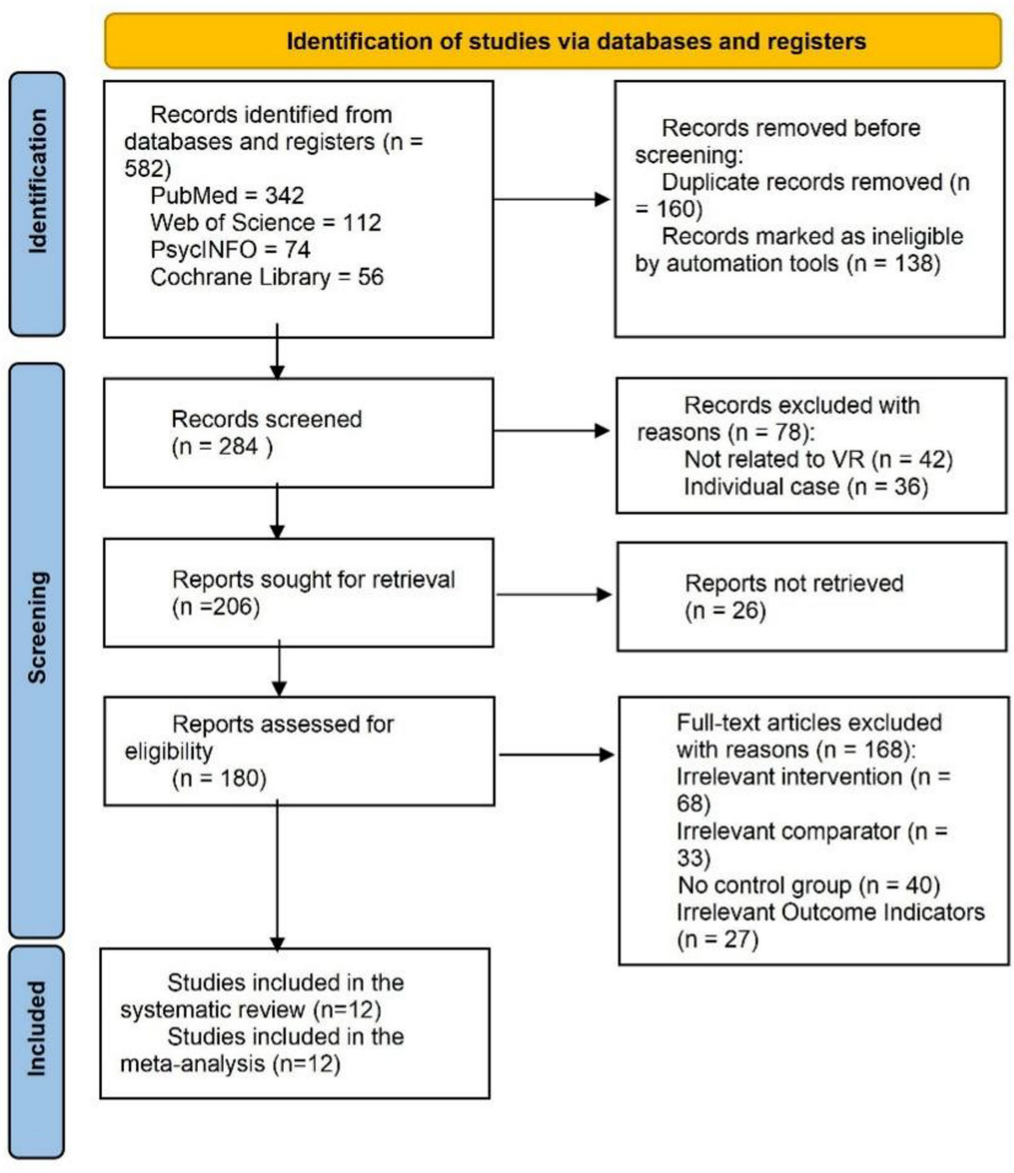
Flow diagram of the selection process.

The initial search across four databases yielded 582 records, of which 160 were duplicates. After screening titles and abstracts of the remaining 422 records, 78 articles were excluded for not meeting the inclusion criteria (Title and Abstract Screening Stage: Cohen’s *κ* = 0.75). The subsequent full-text review of 180 articles excluded 168 articles, with specific reasons outlined in Figure 1. Ultimately, 12 studies were included in this systematic review (Full-Text Screening Stage: Cohen’s *κ* = 0.90).

### Risk of bias of included studies

**Figure 2:**
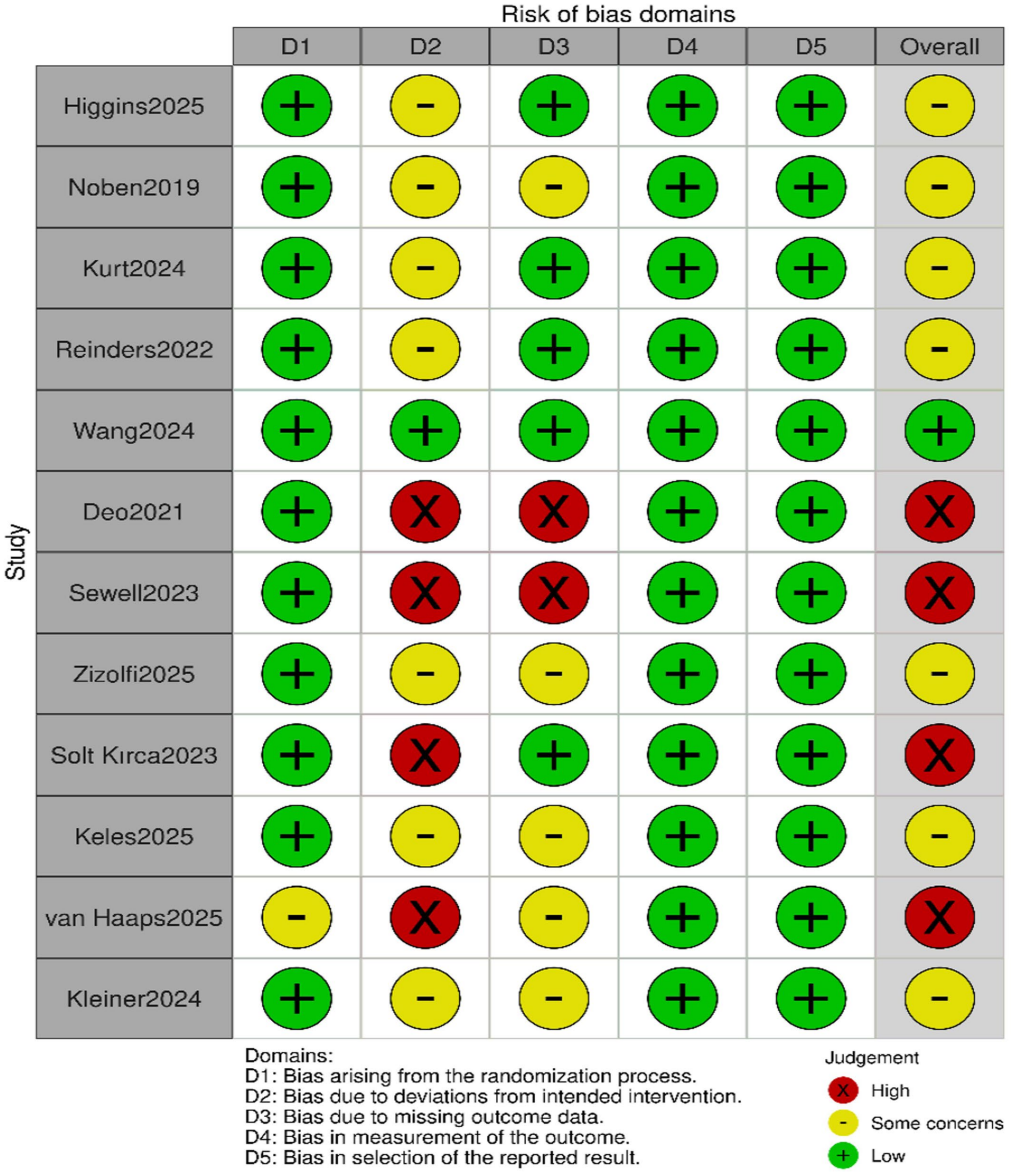
Risk of bias graph.

**Figure 3:**
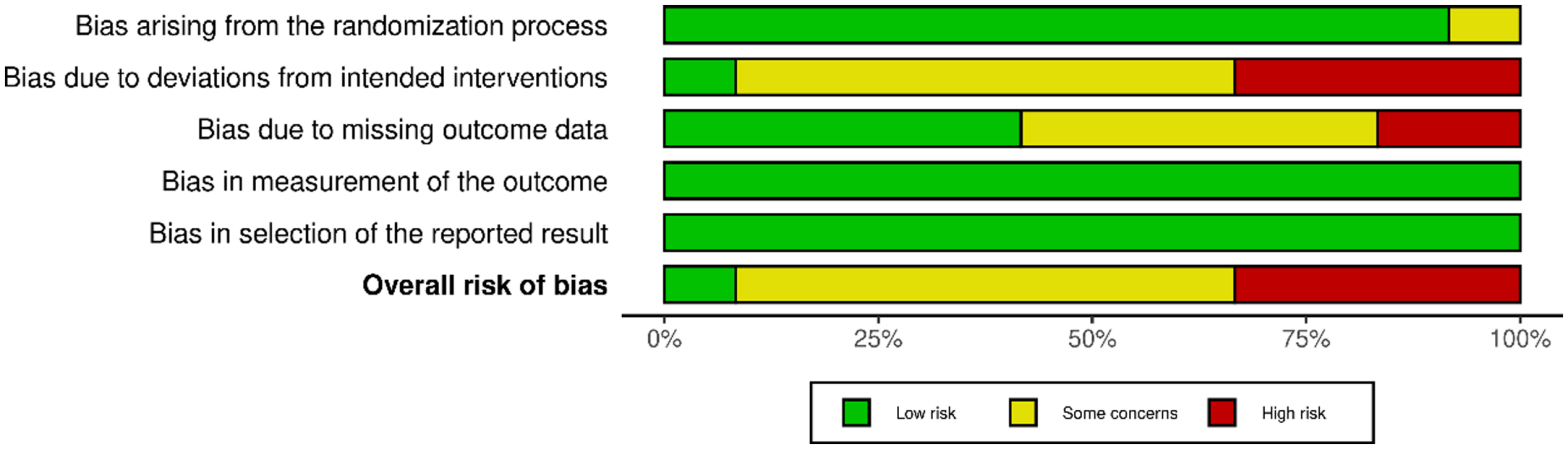
Risk of bias summary.

The results of the risk of bias assessment are presented in Figures 2, 3 (dark orange “X” = high risk, orange “–” = some concerns, light yellow “+” = low risk). Two reviewers independently assessed the risk of bias of the 12 included randomized controlled trials using the five domains of the RoB 2.0 tool (D1: randomization process; D2: deviations from intended interventions; D3: missing outcome data; D4: measurement of the outcome; D5: selection of the reported result). Discrepancies were resolved through discussion, and, when necessary, adjudicated by a third reviewer (see Justifications for judgments and incomplete outcome data for details).

Inter-rater agreement varied across domains. For D1, the simple agreement rate was 83.3% (10/12), with Cohen’s *κ* = −0.091 and weighted *κ* = −0.091. The negative *κ* value may reflect the highly imbalanced distribution of judgments, as most studies were rated as low risk in this domain. For D2 and D3, agreement rates were both 58.3% (7/12), with Cohen’s *κ* values of 0.310 and 0.341, respectively, indicating fair agreement. For D4, the agreement rate was 83.3% (10/12), with Cohen’s *κ* = −0.091 and weighted *κ* = −0.091. Similar to D1, this may be related to the highly imbalanced distribution of ratings. For D5, the agreement rate reached 91.7% (11/12); however, Cohen’s *κ* = 0.000 and weighted *κ* = 0.000, indicating only slight agreement beyond chance.

Most included studies were judged as low risk in the randomization domain. Only one study provided insufficient information on random sequence generation or allocation concealment; therefore, most trials were assessed as having a low risk of bias (van Haaps et al., 2025). Regarding interventions, the inherent nature of VR limited blinding of participants and personnel in 7 studies, posing a potential risk of deviations from the intended interventions. Five studies were assessed as having a low risk of bias due to missing outcome data, as they reported attrition rates and employed Intention-to-Treat (ITT) analysis (Higgins et al., 2025; Kurt and Ozcan 2024; Reinders et al., 2022; Wang et al., 2024; Kırca et al., 2023). The remaining studies raised concerns due to the lack of clear specifications for data-handling methods. Regarding bias in outcome measurement, the methods were relatively objective, and the assessment tools were standardized; therefore, all studies were rated as low risk. All prespecified outcomes were reported, and no evidence of selective reporting was found, indicating a low risk of bias.

### Study characteristics

This study included 12 randomized controlled trials conducted between 2019 and 2025 (Table 1), spanning Australia, the Netherlands, Turkey, China, the United Kingdom, Italy, and Israel. The study participants were predominantly women undergoing gynecological examinations or surgical procedures. These procedures included intrauterine device (IUD) insertion, preoperative preparation for elective Cesarean sections, pelvic examinations, ‘one-stop’ clinic evaluations for abnormal uterine bleeding, gynecological laparoscopic surgeries, outpatient hysteroscopy, episiotomy repairs, and cervical smear tests. Participants’ ages ranged from approximately 20–59 years. All included studies used a randomized, two-arm parallel design (intervention vs. control), with individual study sample sizes ranging from 20 to 66 per group. Regarding interventions, all studies used VR as the primary modality, and some combined VR with standard care or sedation measures. Control groups primarily received routine care, standard care, skin-to-skin contact, or conscious sedation. All interventions were delivered in a single session, lasting approximately 3.25 to 25 min. Anxiety was predominantly assessed using the VAS, STAI-S, HADS-A, and NRS scales.

**Table 1:** Characteristics of the studies in the systematic review and meta-analysis.

Author/year	Country	Design	Sample (T/C)	Age range	Subject Type	Intervention (T/C)	Protocol	Tool
Higgins et al. (2025)	Australia	RCT	36/34	29–43 years	Outpatients undergoing intrauterine device (IUD) insertion	VR vs. Standard Care	12 min/session	VAS
Noben et al. (2019)	Netherlands	RCT	49/48	28–37 years	Pregnant women scheduled for elective cesarean section	VR + standardized procedural information vs. standardized procedural information	4.45 min/session	VAS
Kurt and Ozcan (2024)	Turkey	RCT	64/64	24–37 years	Women undergoing pelvic examination	VR vs. Usual Care	5–7 min/session	STAI-S
Reinders et al. (2022)	Netherlands	RCT	40/43	29–50 years	Women attending a one-stop clinic for abnormal uterine bleeding	VR + Usual Care vs. Usual Care	5.28 min/session	STAI-S
Wang et al. (2024)	China	RCT	58/57	30–45 years	Women undergoing gynecological laparoscopic surgery	VR vs. Standard Care	15 min/session	HADS-A
Deo et al. (2021)	UK	RCT	20/20	25–37 years	Women undergoing outpatient hysteroscopic examination	VR vs. Standard Care	3.25 min/session	NRS
Sewell et al. (2023)	UK	RCT	41/42	37–59 years	Women undergoing outpatient hysteroscopic examination	VR + Standard Care vs. Standard Care	5.5–14.5 min/session	NRS
Zizolfi et al. (2025)	Italy	RCT	58/58	32–58 years	Women undergoing outpatient hysteroscopic examination	VR + Standard Care vs. Standard Care	6.94 min/session	NRS
Kırca et al. (2023)	Turkey	RCT	40/40	20–27 years	Primiparous women undergoing episiotomy repair	VR vs. skin-to-skin vs. usual care	15–25 min/session	STAI-S
Keleş and Toker (2025)	Turkey	RCT	35/35	37–55 years	Women undergoing their first Pap smear examination	VR vs. Standard Care	8–10 min/session	STAI-S
van Haaps et al. (2025)	The Netherlands	RCT	57/56	32–40 years	Women undergoing oocyte retrieval for IVF/ICSI treatment	VR + Standard Conscious Sedation vs. Standard Conscious SedationCare	20.83 min/session	STAI-S
Kleiner et al. (2024)	Israel	RCT	66/66	25–34 years	Pregnant women undergoing induction with transcervical Foley balloon catheter	VR vs. Standard Care	15 min/session	STAI-S

RCT, randomized controlled trial; T/C, Intervention Group/Control Group; min, minutes; VR, Virtual Reality; IUD, Intrauterine Device; IVF, In Vitro Fertilization; ICSI, Intracytoplasmic Sperm Injection; VAS, Visual Analog Scale; STAI-S, State–Trait Anxiety Inventory–State subscale; HADS-A, Hospital Anxiety and Depression Scale–Anxiety subscale; NRS, Numerical Rating Scale.

### Meta-analysis

**Figure 4:**
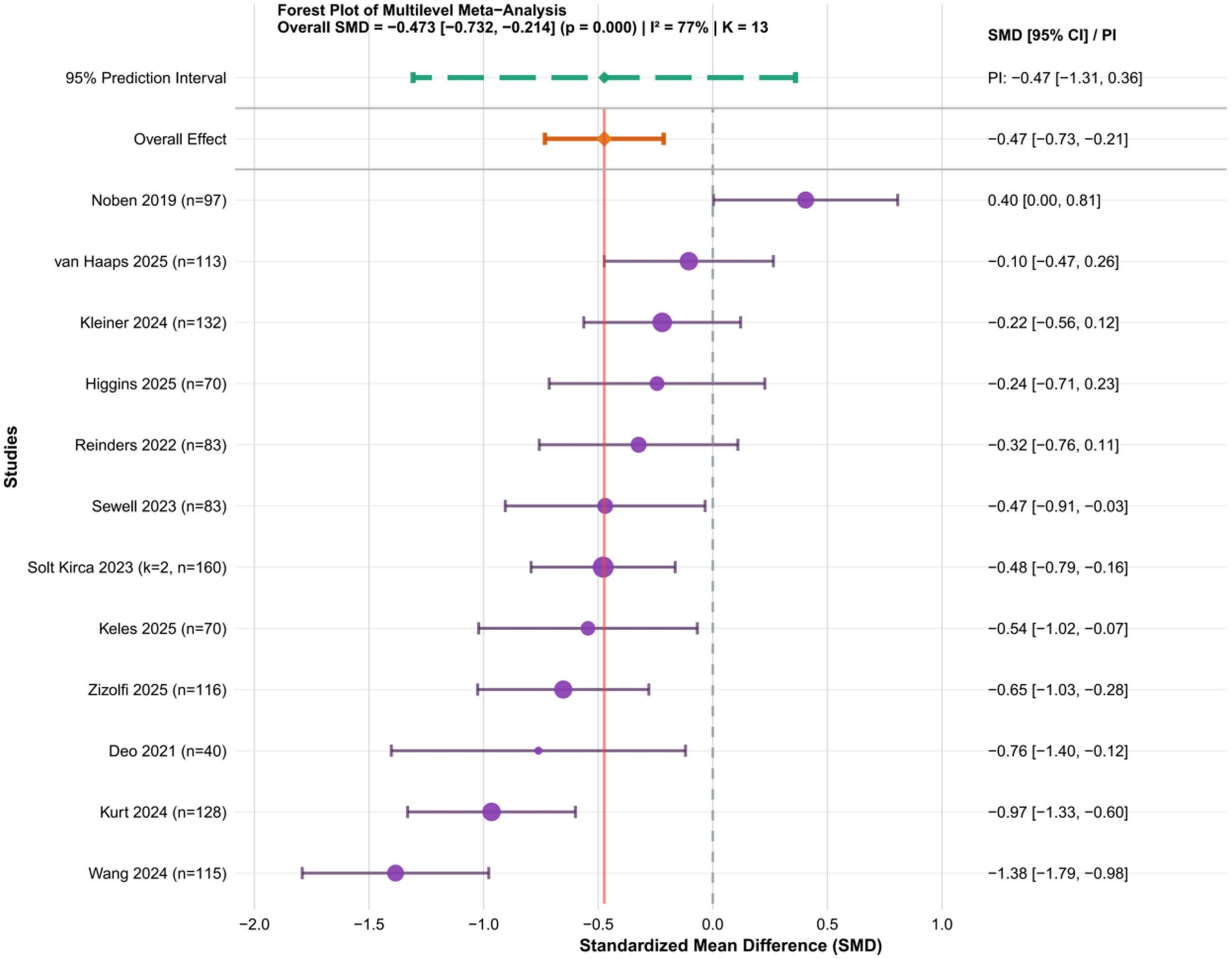
Forest plot of the effect of VR intervention on anxiety symptoms in women undergoing gynecological examinations and surgeries. SMD, standardized mean difference; CI, confidence interval; PI, prediction interval; *I*^2^, heterogeneity statistic; *K*, total number of effect sizes (reflecting the multilevel structure of the data); *N*, number of independent studies; overall effect represents the pooled estimate from a multilevel random-effects model; the 95% prediction interval reflects the dispersion of true effects across studies; negative SMD values indicate reduced anxiety in the VR group relative to controls.

**Figure 5:**
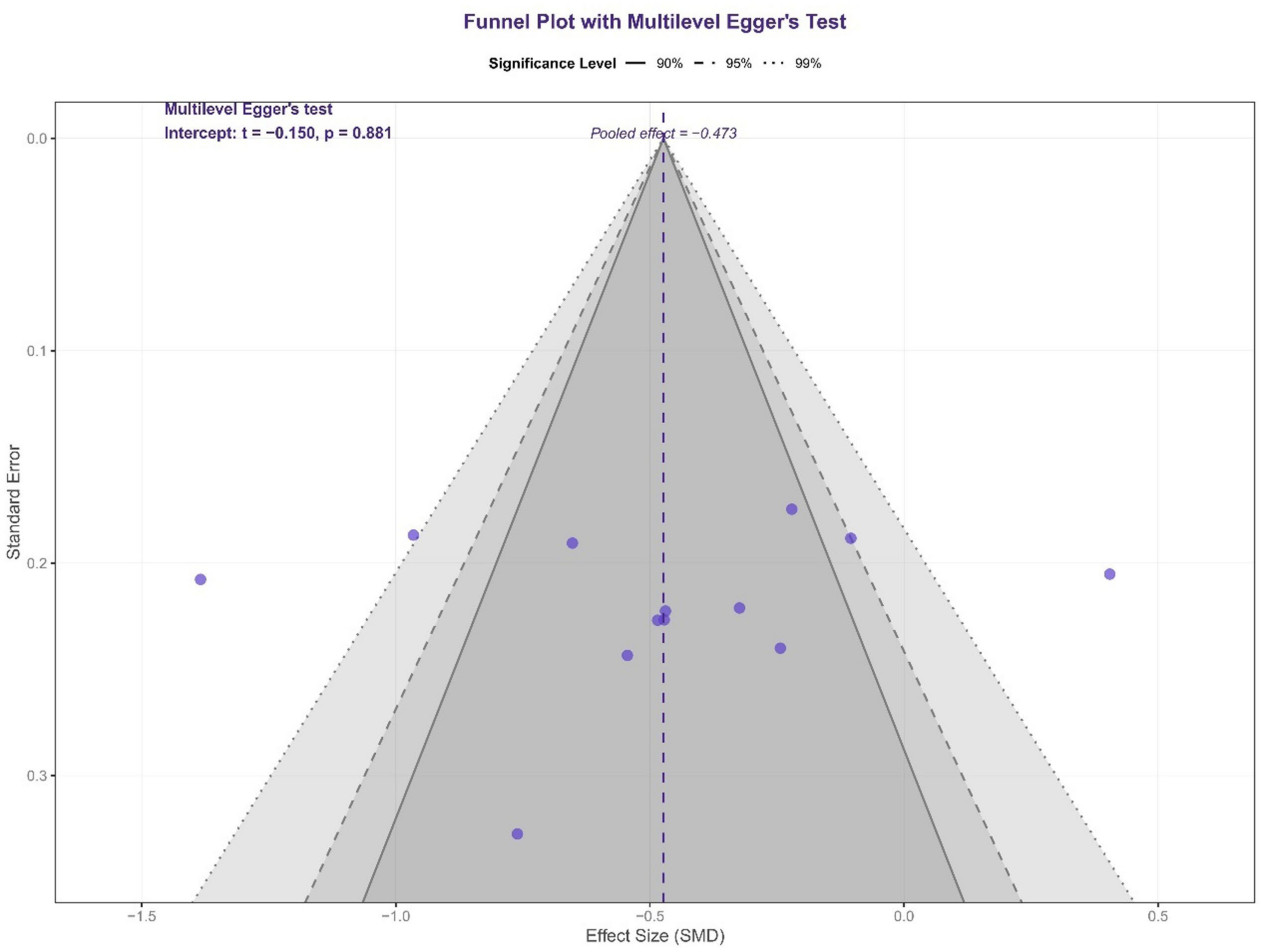
Funnel plots and Egger’s test results for publication bias assessment.

**Figure 6:**
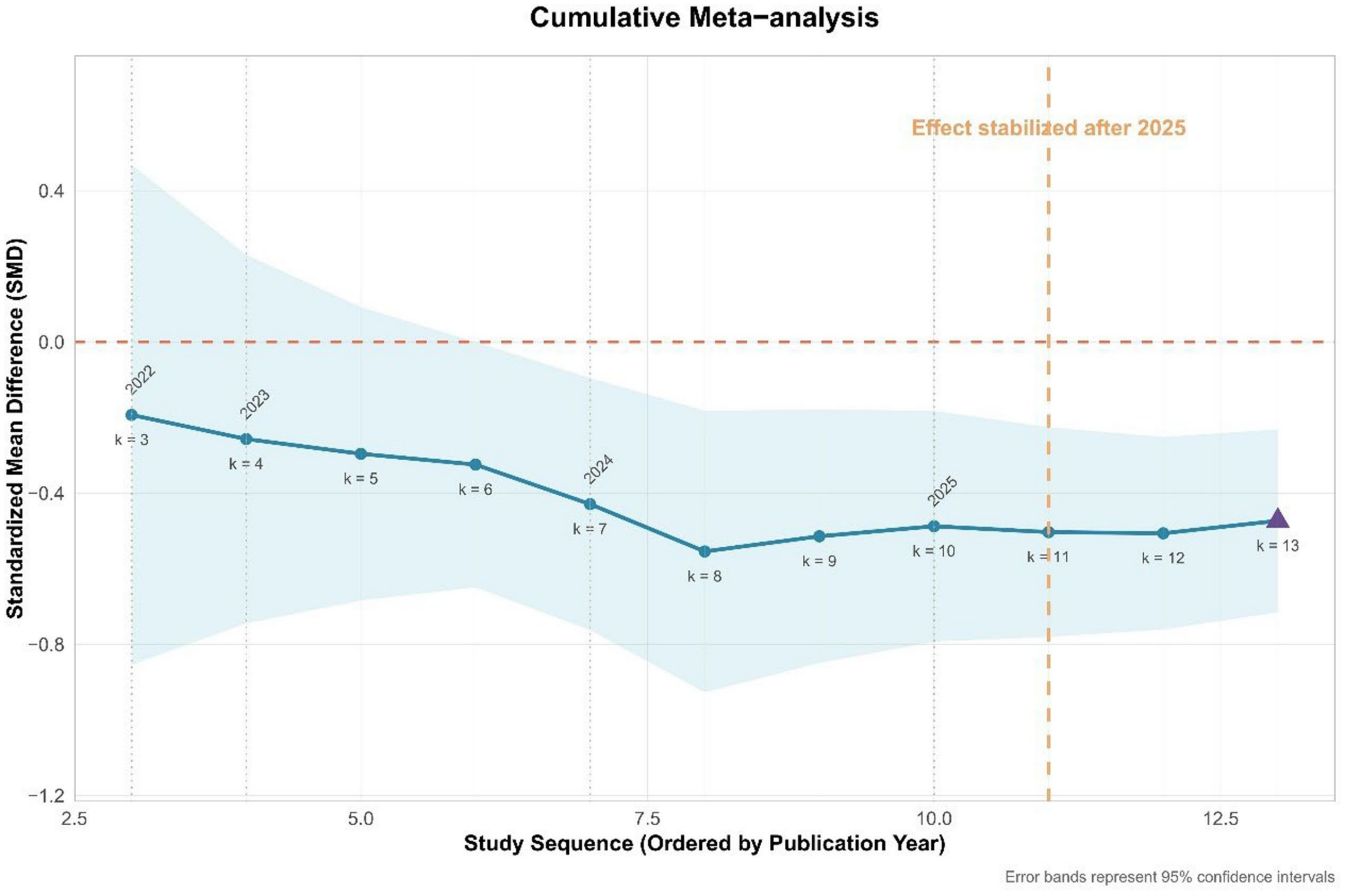
Cumulative meta-analysis.

Twelve studies involving 1,237 participants were included in the meta-analysis. The multilevel model yielded a pooled effect size of −0.47 (95% CI −0.73 to −0.21) (Figure 4), indicating a significant negative effect. However, heterogeneity among studies was substantial (*I*^2^ = 77%, *p* < 0.001). To evaluate publication bias and the influence of individual high-impact studies on the conclusions, a stepwise data-cleaning and diagnostic approach was implemented in accordance with the Cochrane Handbook v6.3. Initially, Egger’s regression, a statistical method for detecting publication bias in meta-analyses, was used to assess whether the results of the studies were affected by small-study effects (Gillespie et al., 2012). The results indicated that no significant publication bias was present during the intervention period (intervention period *t* = −0.150, *p* = 0.881; see Figure 5). Subsequently, statistical diagnostics of the model were conducted using standardized residuals (|*Z*| > 2.5) in conjunction with Cook’s distance (greater than three times the mean) to identify studies that exerted a strong influence and were considered outliers (Viechtbauer and Cheung, 2010). In the intervention endpoint analysis, Wang et al. (2024) and Noben et al. (2019) showed a substantial influence in the Cook’s distance diagnostic; however, their standardized residuals did not exceed the prespecified thresholds, and they were not identified as statistically significant outliers in effect size (see Supplementary material). Sensitivity analyses were subsequently conducted, revealing that after sequentially excluding individual studies, the pooled effect size (SMD) remained stable, ranging from −0.39 to −0.55, and the 95% confidence intervals (CI) for each analysis did not cross the line of no effect. The overall conclusion remained unchanged, thereby verifying the robustness and reliability of the primary analysis results (see Supplementary material). To further assess and adjust for potential publication bias, a reanalysis was performed using the trim-and-fill method, which indicated no missing studies, suggesting a low probability of publication bias and high robustness of the original effect estimates (see Supplementary material). Additionally, to control for random error and assess the sufficiency of the cumulative evidence, a TSA was conducted. The results indicated that the RIS was 42.17, and the current cumulative information size was 151.97, yielding an information ratio of 3.60. The cumulative *Z*-value was −4.79, and its absolute value significantly exceeded the traditional significance threshold (Z-alpha). This indicated that the current cumulative evidence was sufficient, and the overall effect was both statistically significant and stable. Consequently, the likelihood that the further inclusion of new studies would substantially alter the overall conclusion was low (see Figure 6 and Supplementary material). The results of the cumulative meta-analysis, organized by publication year, indicate that as the number of studies gradually increased, the pooled effect size of VR interventions for anxiety symptoms in women undergoing gynecological examinations and surgeries showed a trend toward stabilization. When the number of studies reached *K* = 11 (published in 2025), the pooled effect size had stabilized significantly (SMD = −0.5). Subsequently, as the total number of studies increased to *K* = 13, the final pooled effect size was SMD = −0.47, reflecting a cumulative change of only 0.03. The confidence intervals narrowed further, and the effect direction remained unchanged (see Figure 6). These findings suggest that the current body of evidence on VR interventions for alleviating anxiety symptoms in women undergoing gynecological examinations and surgeries is relatively robust, and that adding further studies of a similar scale is unlikely to significantly alter the overall conclusion. Consequently, the quality of evidence for VR interventions to alleviate anxiety symptoms was assessed using the GRADE approach. Because the included studies were primarily randomized controlled trials, the initial quality of evidence was rated as ‘High’. However, due to significant heterogeneity among the studies (*I*^2^ > 75%), the quality of evidence was downgraded by one level owing to inconsistency. Additionally, although some studies had small sample sizes and there were variations in intervention methods and anxiety measurement tools, these factors did not produce inconsistencies in the direction of the effect. Furthermore, sensitivity analyses, publication bias assessments, and trial sequential analyses all supported the robustness of the results; therefore, the evidence level was not downgraded further and was ultimately rated as moderate-quality evidence (details available in the Supplementary material). To improve the clinical interpretability of the pooled standardized effect, we translated the overall SMD into raw-score units using the VAS 0–10 as an illustrative primary scale. Based on a representative pooled standard deviation from the included VAS studies, the pooled effect of SMD = −0.473 (95% CI: −0.732 to −0.214) corresponded to an estimated −1.32 points (95% CI: −2.04 to −0.60) on the VAS 0–10 scale. When compared with a published MCID of 1 point, the point estimate exceeded the threshold for clinical importance, although the 95% confidence interval did not fully exceed that threshold. The MCID threshold was added to the main forest plot to facilitate clinical interpretation (Table 2).

**Table 2:** Summary of findings and GRADE assessment for anxiety outcomes.

GRADE domain	Judgment	Rationale
Risk of bias	Serious (downgraded 1 level)	Although all included studies were randomized controlled trials, the overall RoB 2 assessment indicated “some concerns” across most studies. Issues were primarily observed in domains D1 (randomization process), D2 (deviations from intended interventions, particularly due to lack of blinding), D3 (missing outcome data), and D5 (selection of reported results). Domain D4 (measurement of the outcome) was consistently rated as low risk. Therefore, the evidence was downgraded by one level due to risk of bias.
Inconsistency	Serious (downgraded 1 level)	Substantial heterogeneity was observed across studies (*I*^2^ = 77%, *p* < 0.001), indicating considerable between-study variability. Although subgroup analyses and meta-regression partially explained heterogeneity, residual inconsistency remained, justifying downgrading for inconsistency.
Indirectness	Not downgraded	The population (women undergoing gynecological examinations and surgeries), intervention (virtual reality), comparators (routine care or standard interventions), and outcome (validated anxiety scales such as STAI-S, HADS-A, VAS, and NRS) were directly aligned with the research question.
Imprecision	Not downgraded	The pooled effect size was statistically significant (SMD = −0.47, 95% CI − 0.73 to −0.21) and did not cross the null value. Although the confidence interval was moderately wide, it consistently indicated a beneficial effect, and TSA confirmed sufficient cumulative evidence.
Publication bias	Not downgraded	Egger’s regression test showed no significant publication bias (*p* = 0.881). Trim-and-fill analysis indicated no missing studies, and sensitivity analyses confirmed the robustness of the results.
Overall quality of evidence	Moderate	The certainty of evidence was initially rated as high because all included studies were RCTs. It was downgraded by one level due to risk of bias and one level due to inconsistency; however, given the robustness of the findings and supporting analyses (sensitivity analysis, TSA), the overall quality of evidence was judged as moderate.

### Subgroup analysis

**Figure 7:**
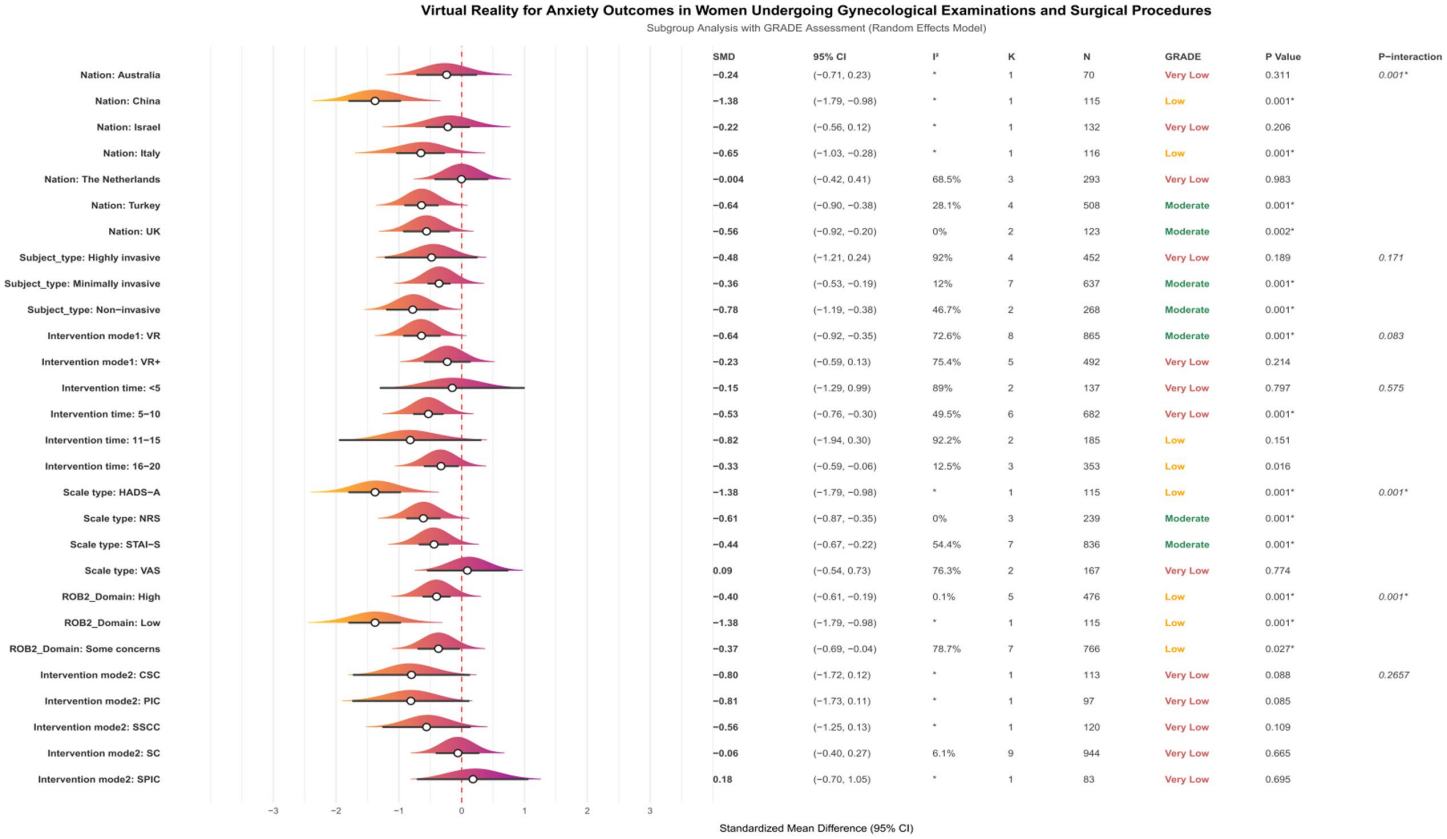
Forest plot of subgroup analysis of the effect of VR intervention on anxiety symptoms in women undergoing gynecological examinations and surgeries. SMD, standardized mean difference; CI, confidence interval; *I*^2^, heterogeneity statistic; *K*, number of studies; *N*, sample size; GRADE, certainty of evidence; ROB2, risk of bias 2; HADS-A, Hospital Anxiety and Depression Scale–Anxiety; STAI-S, State Anxiety Inventory–State; VAS, visual analog scale; NRS, numerical rating scale; VR, virtual reality; VR+, virtual reality plus other interventions; P-interaction, *p*-value for subgroup interaction; CSC, conscious sedation control; PIC, procedural information control; SSCC, skin-to-skin care control; SC, standard care; SPIC, standardized procedural information control.

This study used subgroup analyses to systematically identify potential moderators of the efficacy of VR interventions for anxiety symptoms among women undergoing gynecological examinations and surgeries. The prespecified subgroup variables were: (1) country/region; (2) invasiveness of the procedure; (3) mode of VR intervention; (4) duration of intervention; (5) type of anxiety assessment scale; and (6) methodological quality of the study, as assessed by the RoB 2 tool. The classification of procedural invasiveness (non-invasive, moderately invasive, and highly invasive) was based on an operational definition considering key clinical characteristics, including whether the procedure involved entry into a body cavity, the extent of tissue manipulation, and the requirement for anesthesia or sedation. Non-invasive procedures were defined as those not involving body cavity entry or tissue injury; moderately invasive procedures involved instrument insertion with minimal tissue manipulation; and highly invasive procedures involved substantial tissue manipulation and/or surgical intervention, typically requiring anesthesia. All subgroup analyses were conducted using a random-effects model, and the quality of evidence was systematically evaluated using the GRADE framework (see Figure 7).

Regarding country/region, studies conducted in Turkey (*K* = 4, SMD = −0.64, 95% CI: −0.90 to −0.38, GRADE = Moderate) showed statistically significant anxiety-reducing effects. Consistent with this finding, the subgroups for China (*K* = 1, SMD = −1.38, 95% CI: −1.79 to −0.98), Italy (*K* = 1, SMD = −0.65, 95% CI: −1.03 to −0.28), and the UK (*K* = 2, SMD = −0.56, 95% CI: −0.92 to −0.20) also showed significant treatment effects. In contrast, the subgroups for Israel (*K* = 1, SMD = −0.22, 95% CI: −0.56 to 0.12), Australia (*K* = 1, SMD = −0.24, 95% CI: −0.71 to 0.23), and the Netherlands (*K* = 3, SMD = −0.004, 95% CI: −0.42 to 0.41) did not show a significant effect of VR intervention on anxiety. Among subgroups categorized by invasiveness, VR interventions for non-invasive procedures showed a trend toward a larger effect size (*K* = 2, SMD = −0.78, 95% CI: −1.19 to −0.38, GRADE = Moderate), followed by moderately invasive procedures (*K* = 7, SMD = −0.36, 95% CI: −0.53 to −0.19, GRADE = Moderate). For highly invasive procedures, the effect of VR interventions did not reach statistical significance (*K* = 4, SMD = −0.48, 95% CI: −1.21 to 0.24, *p* = 0.189, *I*^2^ = 92%), indicating no clear evidence of anxiety reduction in this subgroup (see Supplementary Table 2). In the subgroup analysis of Intervention mode 2, no significant difference was observed between subgroups (*P*-interaction = 0.2657). The CSC (*K* = 1, SMD = −0.80, 95% CI: −1.72 to 0.12), PIC (*K* = 1, SMD = −0.81, 95% CI: −1.73 to 0.11), and SSCC (*K* = 1, SMD = −0.56, 95% CI: −1.25 to 0.13) subgroups showed a non-significant trend toward anxiety reduction. In contrast, the SC (*K* = 9, SMD = −0.06, 95% CI: −0.40 to 0.27) and SPIC (*K* = 1, SMD = 0.18, 95% CI: −0.73 to 1.09) subgroups showed no significant effect of VR intervention.

Analysis of intervention modes showed that VR alone exhibited a relatively stable trend in reducing anxiety symptoms (*K* = 8, SMD = −0.64, 95% CI: −0.92 to −0.35, GRADE = Moderate). In contrast, VR combined with other interventions did not reach statistical significance (*K* = 5, SMD = −0.23, 95% CI: −0.59 to 0.13), suggesting that the independent value of VR intervention is likely more pronounced. Regarding intervention duration, the 5–10 min subgroup demonstrated a statistically significant effect (*K* = 6, SMD = −0.53, 95% CI: −0.76 to −0.30, GRADE = Low), suggesting that shorter intervention durations may provide relatively more stable anxiety-reducing outcomes. However, given the limited number of studies and the presence of substantial heterogeneity, this finding should be interpreted with caution. Subgroup analysis across anxiety scales showed that in studies assessing anxiety using the NRS (*K* = 3, SMD = −0.61, GRADE = Moderate), VR intervention outcomes had lower heterogeneity and a relatively stable effect size. When anxiety was assessed using the VAS, the pooled effect was not statistically significant and showed a direction opposite to the overall estimate (*K* = 2, SMD = 0.09, 95% CI: −0.54 to 0.73, *p* = 0.774), indicating no clear evidence of anxiety reduction in this subgroup. In summary, the anxiety-reducing effect of VR intervention was most stable and significant in scenarios involving non-invasive procedures, when VR was used alone, during intervention durations of 5–10 min, and when assessments were conducted using the NRS.

### Meta-regression (linear)

**Figure 8:**
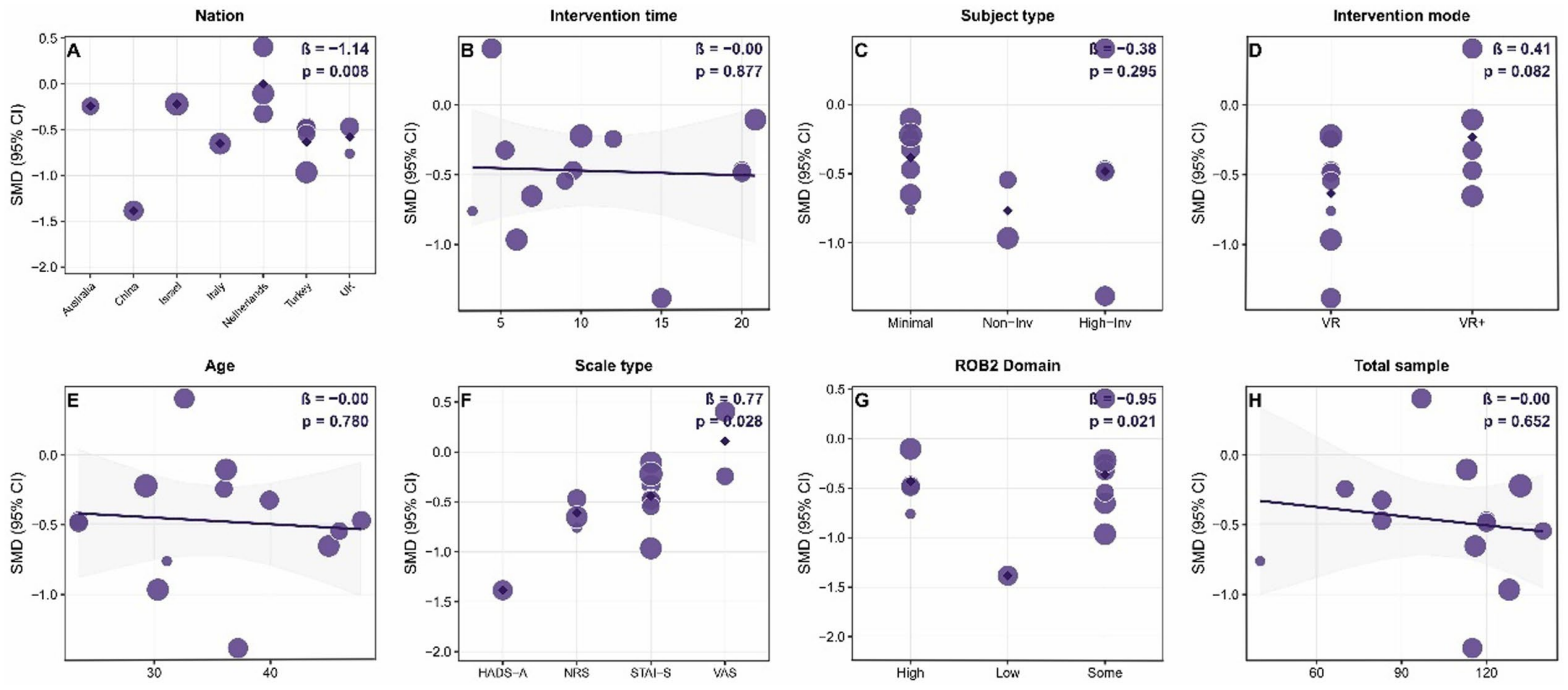
Linear meta-regression bubble plot of the effect of VR intervention on anxiety in women undergoing gynecological examinations and surgeries. **A**: Effect by nation (Australia, China, Iran, Italy, Netherlands, Turkey, UK); **B**: Effect by intervention time (weeks); **C**: Effect by subject type (Minimal, Non-Inv, High-Inv); **D**: Effect by intervention mode (VR, VR+); **E**: Effect by age (years); **F**: Effect by scale type (HADS-A, NRS, STAI-S, VAS); **G**: Effect by ROB2 domain (High, Low, Some); **H**: Overall effect across the total sample.

To further investigate potential sources of between-study heterogeneity, a linear meta-regression analysis was used to systematically assess the relationships between study-level variables and the effect of VR interventions on the improvement of anxiety symptoms among women undergoing gynecological examinations and surgeries (Figure 8). The results indicated a significant linear association between country/region and the intervention effect size (*β* = −1.14, *p* = 0.008), suggesting that national contexts may substantially moderate the efficacy of VR interventions for alleviating anxiety symptoms. Concurrently, the type of anxiety assessment scale showed a significant moderating effect (*β* = 0.77, *p* = 0.028), suggesting that different scales may introduce systematic differences in effect estimates. Furthermore, regression results stratified by ROB2 methodological quality showed a significant correlation between the level of risk of bias and the effect size (*β* = −0.95, *p* = 0.021), suggesting that study quality may influence the magnitude of the VR intervention effect to some extent. In contrast, intervention duration (*β* = −0.00, *p* = 0.877), procedure invasiveness (*β* = −0.38, *p* = 0.295), intervention mode (*β* = 0.41, *p* = 0.082), participant age (*β* = −0.00, *p* = 0.780), and total sample size (*β* = −0.00, *p* = 0.652) did not show significant linear associations with improvement in anxiety symptoms, suggesting that the explanatory power of these variables for the overall effect was limited.

### Nonlinear dose–response modeling

**Figure 9:**
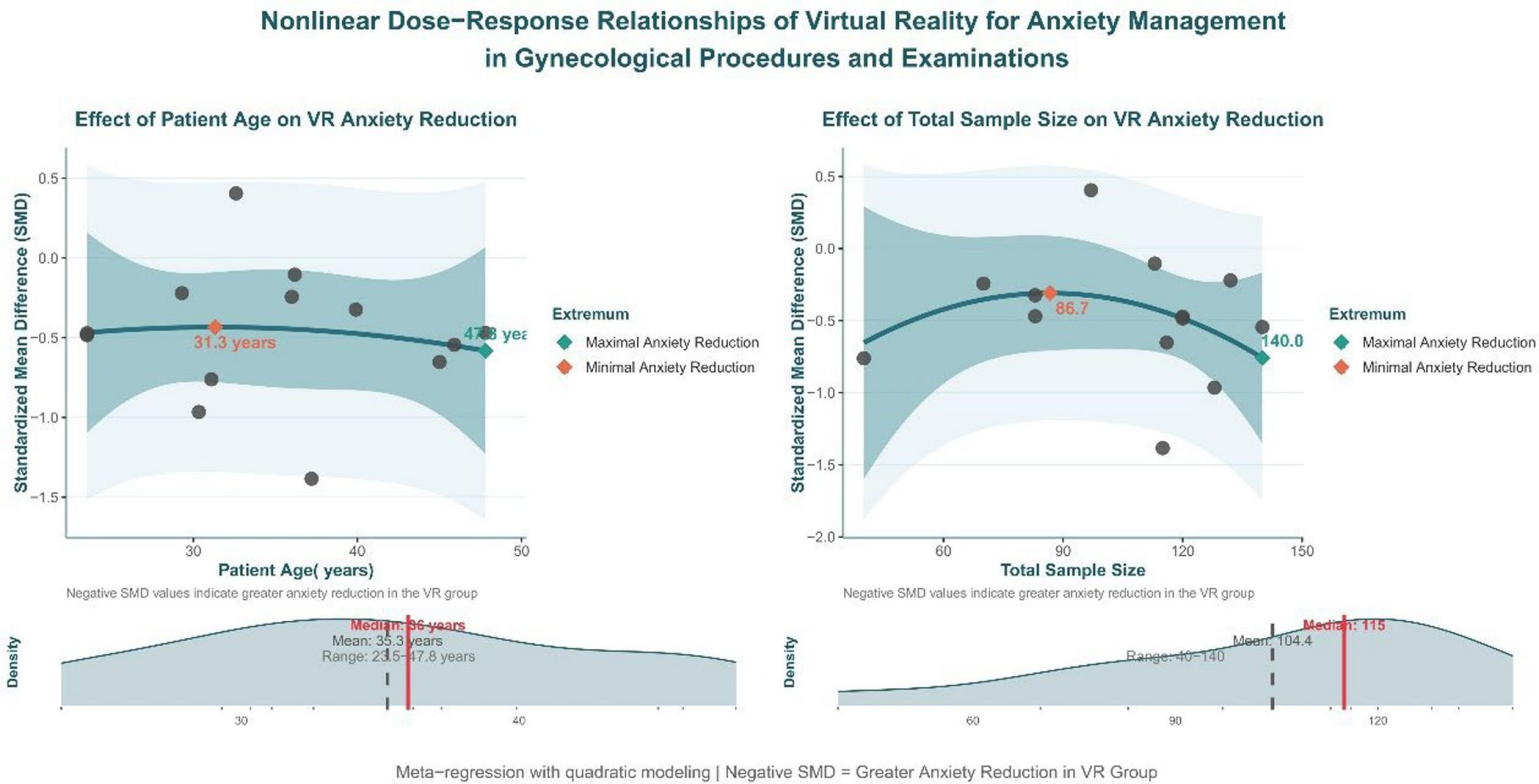
Non-linear meta-regression of the effect of VR intervention on anxiety in women undergoing gynecological examinations and surgeries.

Non-linear analyses suggested potential relationships between VR effects and variables such as age and sample size (see Figure 9). The model suggested a potential peak effect around 47.8 years of age. However, the ages of participants in the included studies were predominantly concentrated between 23.5 and 41.8 years, indicating that the relevant extremum points fell within regions of relatively low sample density. This suggests that the stability of this optimal estimate, based on current evidence, is limited. Therefore, this finding should be regarded as exploratory and should not be directly applied in clinical decision-making until validated by studies with broader age distributions or individual-level data. Potential non-linear patterns were also observed for sample size. The predicted maximum effect point was located at approximately 140 cases, while the minimum effect point was approximately 86.7 cases. Nevertheless, it is important to note that the aforementioned extremum points were located in regions of relatively sparse sample distribution, suggesting that the nonlinear effect of sample size on effect estimation may have been constrained by the uneven distribution of studies, and that the relevant results should be interpreted with caution.

**Figure 10:**
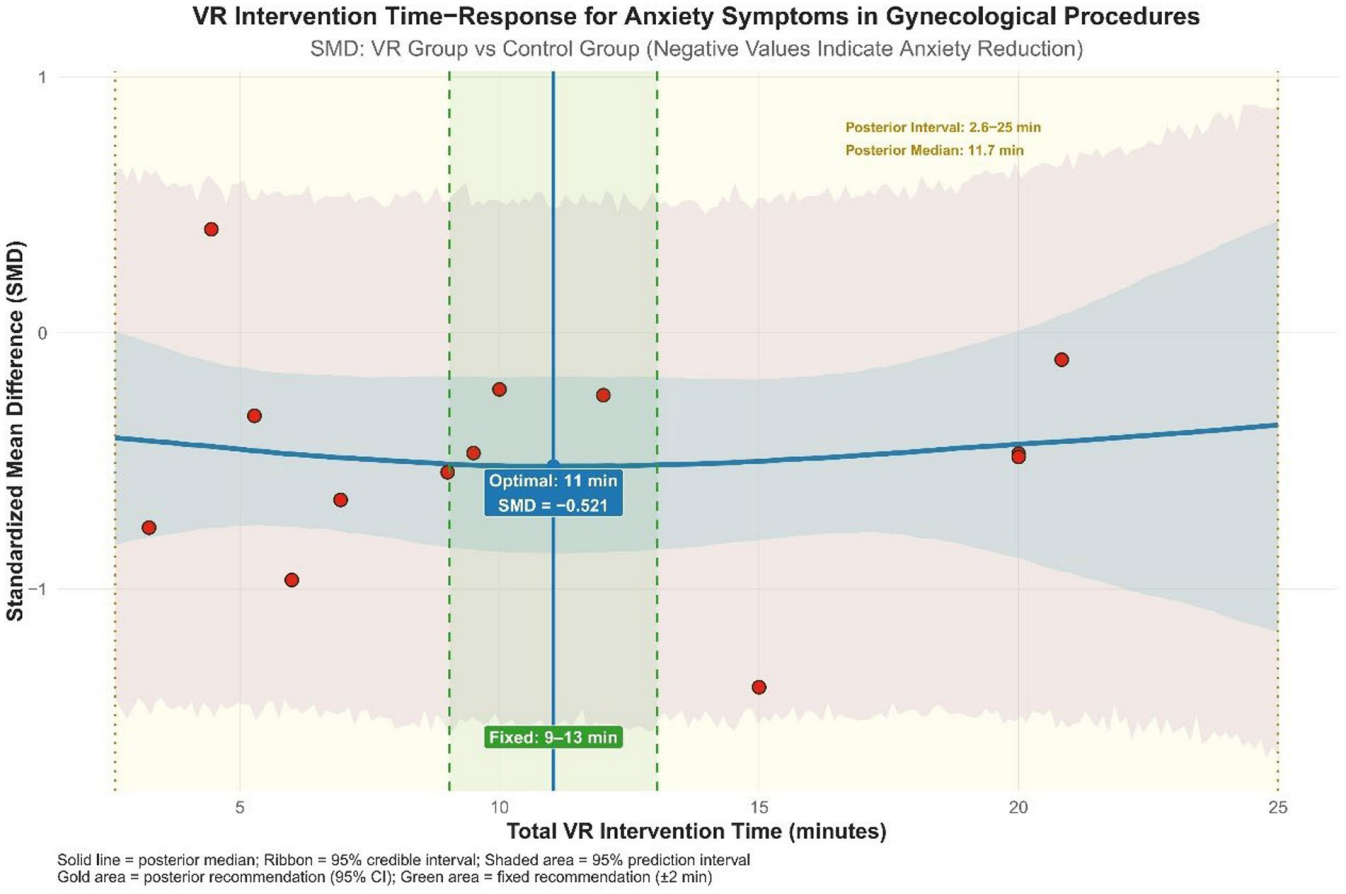
Dose–response relationship between VR intervention and anxiety in women undergoing gynecological examinations and surgeries.

Bayesian dose–response analysis further explored the relationship between intervention duration and anxiety outcomes (see Figure 10). The findings revealed a non-linear association between intervention duration and efficacy. The model predicted an optimal intervention duration of approximately 11.1 min, corresponding to an effect size of SMD = −0.521. A duration range of approximately 9–13 min was identified, although this finding remained exploratory. Within this range, the effect curve remains relatively stable, minimizing uncertainty. Posterior distribution analysis indicated that the median intervention duration is approximately 11.7 min, with a 95% posterior credible interval of 2.6–35 min. However, uncertainty is highest at both ends of this interval, suggesting that further research is needed to validate the optimal dose or intervention duration based on current evidence. Additionally, this study presents posterior samples of the optimal dose (in hours), a global density overlay plot comparing the observed values (*y*ᵢ) with multiple values (*y*_rep_), and an interval plot showing posterior predictive intervals for each observed value against the measured values (see Supplementary material). These findings should be interpreted cautiously because of the limited number of studies and uneven data distribution.

### Summary

This study evaluated the effects of VR interventions on anxiety symptoms among women undergoing gynecological examinations and surgeries. Results showed that VR significantly reduced anxiety symptoms during the intervention period, with a small-to-medium effect size (SMD = −0.47; 95% CI: −0.73 to −0.21; *p* < 0.001). Despite the high heterogeneity observed across the studies (*I*^2^ = 77%), the primary conclusion remained robust after conducting publication bias tests, diagnostics for influential studies, sensitivity analyses, trim-and-fill adjustments, and TSA. The Trial Sequential Analysis showed that the current cumulative information size significantly exceeded the required information size (current information/RSI = 3.60), and the cumulative *Z*-value surpassed the significance threshold, thereby supporting the sufficiency and stability of the overall effect. Subgroup analyses suggested more consistent effects in non-invasive procedures, when VR was administered alone, and when anxiety was assessed using the NRS. Furthermore, linear meta-regression analysis demonstrated that country/region, type of anxiety assessment scale, and methodological quality (RoB 2) were crucial moderators of the effect size. Non-linear analyses suggested potential relationships with age, sample size, and intervention duration. Analysis of intervention duration showed that VR interventions lasting 5–10 min had a statistically significant anxiety-relieving effect. An estimated duration range of approximately 9–13 min was identified; however, this finding should be interpreted as exploratory given the limited data and model uncertainty. Overall, VR was identified as an effective and robust non-pharmacological intervention for anxiety during gynecological examinations and surgery, with its efficacy moderated by patient characteristics, procedure type, and variations in intervention protocols.

## Discussion

Anxiety is a common psychological response during gynecological examinations and surgeries. It not only affects the subjective medical experience but may also hinder cooperation during these procedures and adversely affect postoperative recovery (Huber et al., 2009; Lin et al., 2022). This study systematically integrates results from existing randomized controlled trials and finds that VR interventions significantly reduce anxiety symptoms in women undergoing gynecological examinations and surgeries during the intervention period, with a small-to-medium effect size (SMD = −0.47, 95% CI − 0.73 to −0.21, *p* < 0.001). Although the pooled effect size was expressed as an SMD to allow synthesis across different outcome measures, we additionally translated the pooled estimate into VAS 0–10 units to improve clinical interpretability. This conversion suggested an approximate reduction of 1.32 points, which is larger than the referenced 1-point MCID. However, because the confidence interval ranged from −2.04 to −0.60 points, the uncertainty interval did not remain entirely beyond the MCID threshold. Therefore, the findings suggest that the intervention may be clinically meaningful on average, but this interpretation should be made cautiously. In addition, because the meta-analysis combined multiple instruments, the VAS-based translation should be viewed as an illustrative clinical interpretation rather than a substitute for the primary pooled SMD estimate. This finding aligns with previous studies examining the role of VR in alleviating medical-related anxiety and stress responses, thereby extending the evidence base for its application in gynecological settings (Anderson et al., 2013). Potential mechanisms underlying this effect suggest that VR creates immersive, multisensory environments that engage individuals’ limited attentional resources, thereby reducing their focus on the medical situation and potential threat cues. Consequently, it is a non-pharmacological and highly effective tool for alleviating anxiety and pain across various clinical contexts (Indovina et al., 2018).

The substantial heterogeneity observed in this study likely reflected both methodological and clinical differences across studies. The included procedures varied considerably in invasiveness, procedural complexity, anesthesia requirements, pain expectations, and psychological context, ranging from relatively non-invasive examinations such as Pap smears and pelvic examinations to more invasive procedures including laparoscopic surgery, Cesarean section, and episiotomy repair. Consequently, the underlying mechanisms and intensity of anxiety may differ substantially across these clinical settings. Therefore, although pooling these studies allowed us to examine the overall potential of VR as a non-pharmacological anxiety-reduction strategy in gynecological contexts, the pooled effect size should not be interpreted as uniformly applicable to all procedures. Instead, it should be viewed as representing an overall trend across heterogeneous gynecological settings. The subgroup analyses based on procedural invasiveness were conducted partly to address this issue, and the findings suggested that VR effects may be more stable in less invasive procedures. Nevertheless, residual heterogeneity remained substantial, and future studies focusing on more clinically homogeneous populations are needed to further clarify procedure-specific effects of VR interventions.

Although VR interventions were associated with a statistically significant reduction in anxiety, the presence of substantial heterogeneity suggests that the magnitude of the effect may vary across studies and settings. Consequently, potential sources of heterogeneity were examined alongside the results of the subgroup analyses. Regarding assessment tools, the anxiety-reducing effect of VR showed the greatest stability and significance when evaluated using the NRS (*K* = 3, SMD = −0.61, GRADE = Moderate). Single-item numerical rating tools are particularly suitable for time-constrained clinical settings because of their minimal completion burden and ease of repeated measurement, and they have been shown to be effective in assessing immediate state anxiety (Davey et al., 2007). The NRS, as a single-item numerical tool, has demonstrated strong operationality and measurement validity within clinical populations (Prokopowicz et al., 2022). Previous studies have indicated that the NRS-A and the STAI-State are highly correlated in assessing state anxiety and can distinguish between individuals with high anxiety and those without, suggesting robust validity and feasibility in specific research contexts (Aouad et al., 2016). Notably, the subgroup analysis based on the VAS showed a directionally opposite trend compared with the overall effect, although the result did not reach statistical significance. This finding warrants cautious interpretation. One possible explanation may relate to the measurement characteristics of the VAS itself. As a single-item, immediate self-report tool, the VAS may be more sensitive to transient emotional fluctuations, anticipatory stress, and situational anxiety (Crichton, 2001). In addition, the VAS subgroup included only two studies, and the result appeared to be largely influenced by Noben et al. (2019). Unlike most studies that used VR primarily as a distraction tool during procedures, this study applied a preoperative informational VR video to introduce the surgical process to women scheduled for elective Cesarean section. This type of intervention may have increased participants’ awareness of the upcoming procedure, thereby influencing immediate anxiety ratings. Therefore, the VAS-related finding should not be interpreted as evidence that VR increases anxiety, but rather suggests that differences in anxiety assessment tools, timing of assessment, and the purpose of VR interventions may influence the observed effects. The subgroup analysis of Intervention mode2 suggested that CSC, PIC, and SSCC modalities showed trends toward reducing anxiety, though these findings were each based on single studies (*K* = 1) and thus constitute limited evidence. Notably, the SC subgroup, which had the largest sample size (*K* = 9), showed no significant effect. Overall, the generally inadequate sample sizes across intervention modalities limit the reliability of conclusions, highlighting the need for more high-quality studies to verify the differential effects of various intervention modes.

Regarding intervention modalities, VR interventions alone have shown a significant advantage in alleviating anxiety symptoms (*K* = 8, SMD = −0.64, 95% CI: −0.92 to −0.35, GRADE = Moderate). This outcome may be attributed to the fact that when VR interventions are employed in isolation, their effects are less likely to be confounded by other intervention components, thereby facilitating clearer observation of their independent effects (Chan et al., 2020). Among subgroups with varying levels of invasiveness, VR interventions for non-invasive procedures showed a trend toward a larger effect size (*K* = 2, SMD = −0.78, 95% CI: −1.19 to −0.38, GRADE = Moderate). It should be noted that the classification of procedural invasiveness in this study was based on an operational definition rather than a universally validated framework, which may limit comparability across studies. Research has shown that in non-invasive procedures, such as pelvic examinations, VR, used as a standalone intervention, significantly reduced state anxiety in women (Kurt and Ozcan, 2024). In non-invasive procedures where patients remained fully awake throughout, anxiety was primarily driven by situational factors and anticipation. Consequently, the immersive attentional diversion and emotion-regulation functions of VR were fully utilized, resulting in a more consistent anxiety-relieving effect. Notably, VR interventions did not demonstrate a statistically significant effect in highly invasive procedures. This finding suggests that in the context of major surgical interventions, where anxiety may be driven by factors such as procedural risk, postoperative pain, and pharmacological influences (e.g., anesthesia or sedation), the distraction-based mechanism of VR may be insufficient to meaningfully reduce anxiety. From a clinical perspective, this indicates that VR may be more suitable as an adjunct intervention in less invasive settings, whereas its effectiveness in major surgical contexts remains uncertain. This finding should be interpreted with caution given the limited number of studies and substantial heterogeneity.

Regarding country analysis, studies conducted in Turkey (*K* = 4, SMD = −0.64, 95% CI: −0.90 to −0.38, GRADE = Moderate) showed a statistically significant anxiety-reducing effect. Previous research has indicated that the acceptability and effectiveness of VR may vary across different cultural populations (Riches et al., 2023). Notably, the subgroup analyses for China, Italy, Israel, and Australia were each based on a single study (*K* = 1), indicating that the reliability of these findings is limited and may not be representative of the broader populations in their respective countries. The lack of a significant effect in the Netherlands subgroup warrants further investigation, as it may be associated with specific clinical practice protocols or patient selection biases in that region. Future research with larger, multi-center studies is needed to validate these preliminary findings and to explore how factors such as cultural context and healthcare practice differences may moderate the effects of VR interventions. Non-linear meta-regression and dose–response analyses provided a nuanced perspective on the heterogeneity of VR intervention effects. Although potential nonlinear relationships were observed across the dimensions of age and sample size, the optimal effect points were primarily located in regions with sparse sample distribution. This indicates that the results should be regarded as exploratory findings intended to aid understanding of potential dose–response relationships, rather than as a direct basis for clinical decision-making. Analysis of intervention duration showed that VR interventions lasting 5–10 min had a statistically significant anxiety-relieving effect. The dose–response analysis suggested a potential duration range of approximately 9–13 min. However, given the limited number of included studies, uneven distribution of intervention durations, and model uncertainty, this finding should be considered exploratory rather than a definitive recommendation. Consequently, this finding offers a preliminary reference for future research exploring VR intervention duration settings. Although visual inspection of the funnel plot did not reveal substantial asymmetry, and Egger’s regression test was not statistically significant, it should be noted that with only 12 studies (*K* = 12), the statistical power of such tests is limited. Therefore, the failure to detect publication bias in this analysis does not preclude its existence. The results should be interpreted considering this limitation.

## Conclusion

In summary, VR interventions were associated with a small-to-moderate reduction in anxiety symptoms among women undergoing gynecological examinations and surgeries (SMD = −0.47, 95% CI − 0.73 to −0.21, *p* < 0.001). However, given the substantial heterogeneity across studies, these findings should be interpreted cautiously and may have limited generalisability. The intervention showed a statistically significant overall effect, and the pooled estimate corresponded to an approximate 1.32-point improvement on the VAS 0–10 scale. This value exceeded the referenced MCID of 1 point at the level of the point estimate, suggesting potential clinical relevance. However, because the confidence interval did not fully exceed the MCID threshold, the clinical importance of the effect should be interpreted with caution. The clinical efficacy of these interventions was observed primarily in procedures and implementation conditions that closely matched those of the studies included in this review. Subgroup analyses further indicated that the anxiety-reducing effect of VR was more consistent in non-invasive procedures, when VR was administered alone, and when anxiety was assessed using the Numerical Rating Scale (NRS). Analysis of intervention duration showed that VR interventions lasting 5–10 min had a statistically significant anxiety-relieving effect. Additionally, exploratory Bayesian dose–response analysis suggested that a duration of approximately 9–13 min may represent a potentially relevant range for VR interventions; however, given the limited evidence, this finding remains exploratory and should be interpreted with caution. As a non-pharmacological and readily implementable digital intervention, VR is a promising adjunct to standard care or psychological support. However, its clinical utility requires further validation through rigorous research. Future large-scale multicenter studies are needed to clarify optimal intervention parameters and long-term clinical effects.

### Limitations

This study also presented several limitations that warrant cautious interpretation. First, substantial clinical and methodological heterogeneity was observed across studies, including differences in procedure types, VR protocols, control conditions, and anxiety assessment tools. Despite non-significant subgroup differences, the substantial heterogeneity in control conditions (e.g., standard care, conscious sedation, skin-to-skin contact) may pose limitations for interpreting the pooled effect and generalizing the findings. Second, the analyses were based on study-level aggregate data rather than individual participant data, thereby limiting the extrapolation of conclusions to specific population subgroups. In addition, the classification of procedural invasiveness was based on an operational definition due to the lack of a universally accepted classification system, which may introduce some degree of subjectivity. Third, several studies had relatively small sample sizes, and blinding was difficult because of the nature of VR interventions; this posed a potential risk of bias and lowered the certainty of some evidence. Fourth, most studies focused on short-term outcomes, and evidence regarding long-term effects remains limited. In addition, some included studies did not designate anxiety as the primary outcome, which may have introduced outcome-reporting bias and influenced the pooled estimates. Finally, the exploratory non-linear analyses were limited by the small number of studies and uneven data distribution. Furthermore, although all included studies involved women undergoing gynecological examinations or surgeries, the substantial diversity in procedural characteristics may limit the clinical interpretability of the pooled effect estimate. Therefore, the pooled estimate should be interpreted cautiously across different clinical contexts.

## Data Availability

The original contributions presented in the study are included in the article/Supplementary material, further inquiries can be directed to the corresponding author.
