# Supplementary material for: Effects of virtual reality interventions on anxiety symptoms in women undergoing gynecological examinations and surgeries: a multi-level dose–response meta-analysis
Source: Front Psychol. 2026 May 29;17:1792559. doi: 10.3389/fpsyg.2026.1792559 (PMC13260798; doi:10.3389/fpsyg.2026.1792559)
Supplement: Supplementary file 1 [file Table_1.docx]

**1.ROB2 Assessment for Higgins et al.**

D1 — Bias arising from the randomization process

R1 (Low risk): The study indicates that participants were randomly allocated to intervention groups, suggesting the use of an appropriate random sequence generation method.
R2 (Low risk): Although detailed allocation concealment procedures are not extensively described, there is no indication of imbalance or systematic differences between groups that would suggest compromised allocation.
R3 (Low risk): Baseline characteristics appear comparable across groups, supporting the effectiveness of the randomization process.

Consensus: Low risk
Rationale: The available information supports adequate randomization and baseline comparability, with no clear evidence of selection bias.

D2 — Bias due to deviations from the intended interventions

R1 (Some concerns): The behavioral nature of the VR intervention makes participant blinding impractical, potentially influencing adherence and expectations.

R2 (High risk): The absence of participant blinding, combined with limited information on adherence monitoring, raises concerns about significant deviations from intended interventions.

R3 (Some concerns): Outcome assessors may have been blinded; however, the lack of participant blinding still raises concerns regarding deviations from intended interventions.

Consensus: Some concerns
Rationale: The inability to blind participants in behavioral interventions introduces potential performance bias, although this is partially mitigated by standardized procedures.

D3 — Bias due to missing outcome data

R1 (Low risk): The study appears to report a low level of missing outcome data, suggesting minimal attrition bias.
R2 (Low risk): There is no indication that missing data were related to true outcomes or differed systematically between groups.

Consensus: Low risk
Rationale: The low proportion of missing data and lack of evidence for differential attrition suggest minimal risk of bias in this domain.

D4 — Bias in measurement of the outcome

R1 (Low risk): Outcomes were assessed using standardized and validated measurement tools, reducing the likelihood of measurement error.
R2 (Low risk): Measurement procedures appear consistent across groups, and the nature of the outcomes is unlikely to be influenced by knowledge of intervention assignment.

Consensus: Low risk
Rationale: The use of standardized outcome measures and consistent assessment procedures minimizes the risk of measurement bias.

D5 — Bias in selection of the reported result

R1 (Low risk): The study reports outcomes comprehensively, with no evidence of selective reporting.
R2 (Low risk): Both significant and non-significant results appear to be presented, indicating transparency in reporting.

Consensus: Low risk
Rationale: The consistency between reported outcomes and study objectives suggests a low risk of selective reporting bias.

Overall (Consensus): Some concerns

Rationale: Although most domains are rated as low risk, the presence of some concerns in D2 (deviations from intended interventions) leads to an overall judgment of “some concerns.” The identified limitations do not invalidate the study findings but suggest cautious interpretation.

**2.ROB2 Assessment for Noben et al.**

D1 — Bias arising from the randomization process

R1 (Low risk): The study indicates that participants were randomly assigned to intervention groups, suggesting an appropriate random sequence generation process.
R2 (Low risk): Although detailed allocation concealment procedures are not fully described, there is no evidence of systematic differences between groups that would indicate compromised allocation.
R3 (Low risk): Baseline characteristics appear comparable between groups, supporting the adequacy of the randomization process.

Consensus: Low risk
Rationale: The available information supports appropriate randomization and balanced baseline characteristics, with no indication of selection bias.

D2 — Bias due to deviations from the intended interventions

R1 (High risk): Lack of detailed reporting on intervention adherence and absence of blinding suggest a high likelihood of performance bias.

R2 (Some concerns): Although blinding was not feasible, standardized procedures appear to have been followed, partially mitigating potential bias.

R3 (Some concerns): While outcome assessors may have been blinded, the absence of participant blinding may still affect adherence and behavioral responses.

Consensus: Some concerns
Rationale: The behavioral nature of the intervention and lack of participant blinding introduce some uncertainty regarding deviations from intended interventions.

D3 — Bias due to missing outcome data

R1 (Some concerns): The study does not provide sufficient detail regarding the extent and handling of missing outcome data.
R2 (Some concerns): It is unclear whether missing data were balanced between groups or whether appropriate methods (e.g., imputation) were applied.
R3 (Some concerns): The absence of detailed reporting on attrition and handling of missing data introduces uncertainty regarding potential bias.

Consensus: Some concerns
Rationale: Limited reporting on missing data and its handling results in uncertainty about the potential impact on study outcomes.

D4 — Bias in measurement of the outcome

R1 (Low risk): Outcomes were measured using standardized and validated instruments, ensuring consistency and reliability.
R2 (Low risk): Measurement procedures were likely applied uniformly across groups, reducing the risk of differential measurement bias.

R3 (Low risk): Outcome assessment appears to have been conducted using consistent and validated procedures across groups, making it unlikely that measurement bias substantially influenced the results.

Consensus: Low risk
Rationale: The use of validated measurement tools and consistent procedures minimizes the risk of bias in outcome assessment.

D5 — Bias in selection of the reported result

R1 (Low risk): The study reports outcomes transparently, including both significant and non-significant findings.
R2 (Low risk): There is no evidence suggesting selective outcome reporting.

R3 (Low risk): Reported outcomes appear complete and aligned with the study objectives, with no indication of selective omission or emphasis.

Consensus: Low risk
Rationale: The completeness and transparency of reporting indicate a low risk of selective reporting bias.

Overall (Consensus): Some concerns

Rationale: Although most domains are rated as low risk, the presence of some concerns in D2 (deviations from intended interventions) and D3 (missing outcome data) results in an overall judgment of “some concerns.” These limitations suggest cautious interpretation but do not undermine the validity of the study findings.

**3.ROB2 Assessment for Kurt et al.**

D1 — Bias arising from the randomization process

R1 (Low risk): Randomization procedures appear appropriate, with no indication of selection bias.

R2 (Some concerns): Insufficient detail regarding allocation concealment introduces some uncertainty in the randomization process.

R3 (Low risk): Baseline characteristics appear comparable across groups, supporting the adequacy of the randomization process.

Consensus: Low risk
Rationale: The available information supports appropriate randomization and balanced baseline characteristics, with no evidence of selection bias.

D2 — Bias due to deviations from the intended interventions

R1 (Some concerns): Lack of participant blinding may have influenced intervention adherence.

R2 (Low risk): The use of structured protocols likely minimized deviations from intended interventions.

R3 (Some concerns): While outcome assessment procedures may have been controlled, lack of participant blinding may still affect adherence and behavioral responses.

Consensus: Some concerns
Rationale: The behavioral nature of the intervention and lack of participant blinding introduce uncertainty regarding deviations from intended interventions.

D3 — Bias due to missing outcome data

R1 (Low risk): The study appears to report low levels of missing outcome data, suggesting minimal attrition bias.
R2 (Low risk): There is no indication that missing data were related to true outcomes or differed systematically between groups.

R3 (Low risk): The proportion of missing outcome data is likely insufficient to meaningfully influence the study results, and no evidence suggests differential attrition between groups.

Consensus: Low risk
Rationale: The low proportion of missing data and absence of evidence for differential attrition indicate minimal risk of bias in this domain.

D4 — Bias in measurement of the outcome

R1 (Low risk): Outcomes were assessed using standardized and validated instruments, ensuring measurement reliability.
R2 (Low risk): Measurement procedures appear to have been applied consistently across groups, reducing the likelihood of differential measurement bias.

R3 (Low risk): The outcome assessment methods appear appropriate and consistently implemented, and it is unlikely that knowledge of intervention assignment substantially influenced the measurement process.

Consensus: Low risk
Rationale: The use of validated tools and consistent assessment methods minimizes measurement bias.

D5 — Bias in selection of the reported result

R1 (Low risk): The study reports outcomes comprehensively, including both significant and non-significant findings.
R2 (Low risk): There is no evidence of selective outcome reporting.

R3 (Low risk): Reported outcomes appear consistent with the study objectives and expected analyses, with no indication that results were selectively omitted or selectively emphasized.

Consensus: Low risk
Rationale: Transparent and complete reporting indicates a low risk of selective reporting bias.

Overall (Consensus): Some concerns

Rationale: Although most domains are rated as low risk, the presence of some concerns in D2 (deviations from intended interventions) leads to an overall judgment of “some concerns.” These limitations suggest cautious interpretation but do not compromise the overall validity of the findings.

**4.ROB2 Assessment for Reinders et al.**

D1 — Bias arising from the randomization process

R1 (Low risk): The study reports that participants were randomly assigned to intervention groups, indicating the use of an appropriate random sequence generation method.
R2 (Low risk): Although allocation concealment procedures are not described in detail, there is no indication of systematic imbalance between groups.
R3 (Low risk): Baseline characteristics appear comparable across groups, supporting the adequacy of the randomization process.

Consensus: Low risk
Rationale: Adequate randomization and balanced baseline characteristics suggest minimal risk of selection bias.

D2 — Bias due to deviations from the intended interventions

R1 (Some concerns): The intervention involved a virtual reality–based approach, and participant blinding was not feasible, which may influence expectations and engagement.
R2 (Some concerns): Although intervention delivery was likely standardized, the absence of participant blinding introduces potential performance bias.
R3 (Some concerns): While outcome assessment procedures may have been controlled, lack of participant blinding may still affect adherence and behavioral responses.

Consensus: Some concerns
Rationale: The behavioral nature of the intervention and lack of participant blinding introduce uncertainty regarding deviations from intended interventions.

D3 — Bias due to missing outcome data

R1 (Low risk): The study reports a low level of missing outcome data, suggesting minimal attrition bias.
R2 (Low risk): There is no indication that missing data were related to outcomes or differed between groups.

R3 (Low risk): The extent of missing outcome data appears limited, and there is no evidence suggesting that attrition was systematic or outcome-dependent.

Consensus: Low risk
Rationale: The low proportion of missing data and absence of differential attrition indicate minimal risk of bias.

D4 — Bias in measurement of the outcome

R1 (Low risk): Outcomes were assessed using standardized and validated instruments, ensuring consistency and reliability.
R2 (Low risk): Measurement procedures appear to have been applied consistently across groups, reducing the likelihood of differential measurement bias.

R3 (Low risk): Outcome assessment methods appear appropriate and consistently implemented, and it is unlikely that measurement was influenced by knowledge of group allocation.

Consensus: Low risk
Rationale: The use of validated tools and standardized procedures minimizes bias in outcome assessment.

D5 — Bias in selection of the reported result

R1 (Low risk): The study reports outcomes comprehensively, including both significant and non-significant findings.
R2 (Low risk): There is no evidence of selective outcome reporting.

R3 (Low risk): Reported outcomes appear consistent with the study objectives and expected analyses, with no indication of selective omission or reporting bias.

Consensus: Low risk
Rationale: Transparent and complete reporting indicates a low risk of selective reporting bias.

Overall (Consensus): Some concerns

Rationale: Although most domains are rated as low risk, the presence of some concerns in D2 (deviations from intended interventions) results in an overall judgment of “some concerns.” These limitations suggest cautious interpretation but do not substantially undermine the validity of the findings.

**5.ROB2 Assessment for Wang et al.**

D1 — Bias arising from the randomization process

R1 (Low risk): The study reports that participants were randomly assigned to intervention groups, indicating the use of an appropriate and standardized random sequence generation method.
R2 (Low risk): Allocation procedures appear to have been adequately implemented, with no indication of compromised allocation concealment or selection bias.
R3 (Low risk): Baseline characteristics are well balanced between groups, supporting the effectiveness of the randomization process.

Consensus: Low risk
Rationale: Clear evidence of appropriate randomization and balanced baseline characteristics suggests minimal risk of bias arising from the randomization process.

D2 — Bias due to deviations from the intended interventions

R1 (Low risk): The intervention protocol appears to have been clearly defined and consistently implemented across participants.
R2 (Low risk): There is no indication of substantial deviations from the intended intervention that would systematically differ between groups.
R3 (Low risk): The study design and reporting suggest that any deviations were minimal and unlikely to influence outcomes.

Consensus: Low risk
Rationale: The study demonstrates good adherence to the intended intervention with no evidence of performance bias affecting the results.

D3 — Bias due to missing outcome data

R1 (Low risk): The study reports minimal missing outcome data, indicating a low level of attrition.
R2 (Low risk): There is no evidence that missing data were related to the outcomes or differed between groups.
R3 (Low risk): Appropriate handling of missing data appears to have been applied or the impact is negligible due to low attrition.

Consensus: Low risk
Rationale: The low level of missing data and absence of differential attrition suggest minimal risk of bias in this domain.

D4 — Bias in measurement of the outcome

R1 (Low risk): Outcomes were measured using standardized and validated assessment tools.
R2 (Low risk): Measurement procedures appear to have been applied consistently across groups, reducing the risk of measurement bias.
R3 (Low risk): Outcome assessment is unlikely to have been influenced by knowledge of intervention allocation.

Consensus: Low risk
Rationale: The use of standardized instruments and consistent procedures ensures reliable outcome measurement.

D5 — Bias in selection of the reported result

R1 (Low risk): The study reports outcomes comprehensively and transparently.
R2 (Low risk): There is no evidence of selective reporting of outcomes.

R3 (Low risk): Reported outcomes appear complete and consistent with the study objectives, with no indication of selective omission or preferential reporting.

Consensus: Low risk
Rationale: Complete and consistent reporting indicates a low risk of selective reporting bias.

Overall (Consensus): Low risk

Rationale: All domains are rated as low risk, indicating a high level of methodological rigor. The study provides robust and reliable evidence with minimal risk of bias across all domains.

**6.ROB2 Assessment for Deo et al.**

D1 — Bias arising from the randomization process

R1 (Low risk): The study reports that participants were randomly assigned to intervention groups, suggesting the use of an appropriate random sequence generation method.
R2 (Low risk): Although detailed allocation concealment procedures are not explicitly described, there is no indication of systematic imbalance between groups.
R3 (Low risk): Baseline characteristics appear comparable across groups, supporting the adequacy of the randomization process.

Consensus: Low risk
Rationale: The available information suggests appropriate randomization and balanced baseline characteristics, indicating a low risk of selection bias.

D2 — Bias due to deviations from the intended interventions

R1 (High risk): The intervention involved a behavioral virtual reality–based approach, and no evidence of participant or personnel blinding was reported, which may substantially influence participant expectations and behaviors.
R2 (High risk): There is insufficient information regarding adherence monitoring or whether deviations from the intended intervention were balanced between groups.
R3 (High risk): The lack of blinding combined with limited reporting on protocol fidelity increases the likelihood that deviations from intended interventions could have influenced the outcomes.

Consensus: High risk
Rationale: The absence of blinding and insufficient control of intervention fidelity introduce a substantial risk of performance bias.

D3 — Bias due to missing outcome data

R1 (High risk): The study provides limited information regarding the extent and handling of missing outcome data.
R2 (High risk): There is no clear description of methods used to address missing data (e.g., ITT analysis, imputation), raising concerns about potential attrition bias.
R3 (High risk): Missing data may be related to outcomes or differ between groups, which could bias the effect estimates.

Consensus: High risk
Rationale: Insufficient reporting and lack of appropriate handling of missing data suggest a high risk of attrition bias.

D4 — Bias in measurement of the outcome

R1 (Low risk): Outcomes were assessed using standardized and validated measurement tools.
R2 (Low risk): Measurement procedures appear to have been applied consistently across groups, reducing the likelihood of differential measurement bias.

R3 (Low risk): Outcome assessment methods appear appropriate and consistently implemented, and it is unlikely that measurement was substantially influenced by knowledge of intervention assignment.

Consensus: Low risk
Rationale: The use of validated instruments and consistent assessment procedures minimizes measurement bias.

D5 — Bias in selection of the reported result

R1 (Low risk): The study reports outcomes comprehensively, including both significant and non-significant findings.
R2 (Low risk): There is no indication of selective outcome reporting.

R3 (Low risk): Reported outcomes appear complete and consistent with the study objectives, with no evidence of selective omission or preferential reporting.

Consensus: Low risk
Rationale: Transparent reporting suggests a low risk of selective reporting bias.

Overall (Consensus): High risk

Rationale: Although D1, D4, and D5 are rated as low risk, the presence of high risk in D2 (deviations from intended interventions) and D3 (missing outcome data) leads to an overall judgment of “high risk.” These limitations may substantially affect the internal validity of the study and should be considered when interpreting the pooled results.

**7.ROB2 Assessment for Sewell et al.**

D1 — Bias arising from the randomization process

R1 (Low risk): The study reports that participants were randomly assigned to intervention groups, indicating the use of an appropriate random sequence generation method.
R2 (Low risk): Although allocation concealment procedures are not described in detail, there is no indication of systematic imbalance between groups.
R3 (Low risk): Baseline characteristics appear comparable across groups, supporting the adequacy of the randomization process.

Consensus: Low risk
Rationale: Adequate randomization and balanced baseline characteristics suggest minimal risk of selection bias.

D2 — Bias due to deviations from the intended interventions

R1 (High risk): The intervention involved a virtual reality–based approach, and no participant blinding was reported, which may substantially influence participant expectations and behavioral responses.
R2 (High risk): There is limited information on adherence monitoring or whether deviations from intended interventions were controlled or balanced between groups.
R3 (High risk): The lack of blinding combined with insufficient reporting of protocol fidelity increases the likelihood that deviations from intended interventions may have affected outcomes.

Consensus: High risk
Rationale: The absence of participant blinding and insufficient control of intervention implementation introduce a substantial risk of performance bias.

D3 — Bias due to missing outcome data

R1 (High risk): The study provides limited information regarding the extent of missing outcome data.
R2 (High risk): There is no clear description of how missing data were handled (e.g., ITT analysis or imputation methods), raising concerns about potential attrition bias.
R3 (High risk): Missing data may not be random and could influence the observed effect estimates.

Consensus: High risk
Rationale: Insufficient reporting and lack of appropriate handling of missing data suggest a high risk of attrition bias.

D4 — Bias in measurement of the outcome

R1 (Some concerns): Outcome assessment may have been influenced by lack of assessor blinding.

R2 (Low risk): The use of validated measurement tools and standardized procedures likely minimized measurement bias.

R3 (Low risk): Although assessor blinding was not clearly reported, the use of validated instruments and consistent assessment procedures likely reduced the risk of measurement bias.

Consensus: Low risk
Rationale: The use of validated tools and consistent assessment procedures minimizes measurement bias.

D5 — Bias in selection of the reported result

R1 (Low risk): The study reports outcomes comprehensively, including both significant and non-significant findings.
R2 (Low risk): There is no indication of selective outcome reporting.

R3 (Low risk): Reported outcomes appear complete and aligned with the study objectives, with no evidence of selective omission or preferential reporting.

Consensus: Low risk
Rationale: Transparent and complete reporting indicates a low risk of selective reporting bias.

Overall (Consensus): High risk

Rationale: Although D1, D4, and D5 are rated as low risk, the presence of high risk in D2 (deviations from intended interventions) and D3 (missing outcome data) results in an overall judgment of “high risk.” These methodological limitations may substantially affect the internal validity of the study and should be considered when interpreting the pooled results.

**8.ROB2 Assessment for Zizolfi et al.**

D1 — Bias arising from the randomization process

R1 (Low risk): The study reports that participants were randomly assigned to intervention groups, indicating the use of an appropriate random sequence generation method.
R2 (Low risk): Although detailed allocation concealment procedures are not explicitly described, there is no indication of systematic imbalance between groups.
R3 (Low risk): Baseline characteristics appear comparable across groups, supporting the adequacy of the randomization process.

Consensus: Low risk
Rationale: Adequate randomization and balanced baseline characteristics suggest minimal risk of selection bias.

D2 — Bias due to deviations from the intended interventions

R1 (Some concerns): Participant blinding was not feasible, introducing potential performance bias.

R2 (Some concerns): Despite lack of blinding, intervention delivery appears consistent across groups.

R3 (Some concerns): While outcome assessment procedures may have been controlled, lack of participant blinding may still affect adherence and behavioral responses.

Consensus: Some concerns
Rationale: The behavioral nature of the intervention and lack of participant blinding introduce uncertainty regarding deviations from intended interventions.

D3 — Bias due to missing outcome data

R1 (Some concerns): The study reports outcome data, but detailed information regarding the extent and reasons for missing data is limited.
R2 (Some concerns): There is no clear description of methods used to handle missing data (e.g., intention-to-treat analysis or imputation techniques).
R3 (Some concerns): It is unclear whether missing data may be related to outcomes, introducing potential attrition bias.

Consensus: Some concerns
Rationale: Limited reporting on missing data and handling methods introduces uncertainty regarding the potential impact of attrition on study results.

D4 — Bias in measurement of the outcome

R1 (Low risk): Outcomes were assessed using standardized and validated measurement tools.
R2 (Low risk): Measurement procedures appear to have been applied consistently across groups, reducing the likelihood of differential measurement bias.

R3 (Low risk): Outcome assessment methods appear appropriate and consistently implemented, and it is unlikely that measurement was influenced by knowledge of group allocation.

Consensus: Low risk
Rationale: The use of validated instruments and consistent measurement procedures minimizes bias in outcome assessment.

D5 — Bias in selection of the reported result

R1 (Low risk): The study reports outcomes comprehensively, including both significant and non-significant findings.
R2 (Low risk): There is no indication of selective outcome reporting.

R3 (Low risk): Reported outcomes appear complete and consistent with the study objectives, with no evidence of selective omission or reporting bias.

Consensus: Low risk
Rationale: Transparent and complete reporting indicates a low risk of selective reporting bias.

Overall (Consensus): Some concerns

Rationale: Although several domains are rated as low risk, the presence of some concerns in both D2 (deviations from intended interventions) and D3 (missing outcome data) leads to an overall judgment of “some concerns.” These limitations suggest cautious interpretation but do not substantially compromise the validity of the study findings.

**9.ROB2 Assessment for Şolt Kırca et al.**

D1 — Bias arising from the randomization process

R1 (Low risk): The study reports that participants were randomly allocated to intervention groups, indicating the use of an appropriate random sequence generation method.
R2 (Low risk): Although allocation concealment procedures are not explicitly described, there is no evidence of systematic imbalance between groups.
R3 (Low risk): Baseline characteristics appear comparable across groups, supporting the adequacy of the randomization process.

Consensus: Low risk
Rationale: The available information supports appropriate randomization and balanced baseline characteristics, suggesting minimal selection bias.

D2 — Bias due to deviations from the intended interventions

R1 (High risk): Significant concerns arise due to lack of blinding and unclear adherence to intervention protocols.

R2 (Some concerns): Although blinding was absent, standardized procedures may have reduced the impact of deviations.

R3 (High risk): The lack of participant blinding and limited reporting on intervention fidelity increase the likelihood that deviations from intended interventions may have affected outcomes.

Consensus: High risk
Rationale: The absence of participant blinding and insufficient control of intervention delivery introduce a substantial risk of performance bias.

D3 — Bias due to missing outcome data

R1 (Low risk): The study appears to report a low level of missing outcome data.
R2 (Low risk): There is no indication that missing data were related to outcomes or differed systematically between groups.

R3 (Low risk): The extent of missing outcome data appears limited, and there is no evidence suggesting that attrition was systematic or outcome-dependent.

Consensus: Low risk
Rationale: The low proportion of missing data and absence of differential attrition indicate minimal risk of bias.

D4 — Bias in measurement of the outcome

R1 (Low risk): Outcomes were assessed using standardized and validated instruments.
R2 (Low risk): Measurement procedures appear to have been applied consistently across groups, reducing the likelihood of differential measurement bias.

R3 (Low risk): Outcome assessment methods appear appropriate and consistently implemented, and it is unlikely that measurement was influenced by knowledge of group allocation.

Consensus: Low risk
Rationale: The use of validated tools and consistent assessment procedures minimizes measurement bias.

D5 — Bias in selection of the reported result

R1 (Low risk): The study reports outcomes comprehensively, including both significant and non-significant findings.
R2 (Low risk): There is no evidence of selective outcome reporting.

R3 (Low risk): Reported outcomes appear complete and consistent with the study objectives, with no indication of selective omission or preferential reporting.

Consensus: Low risk
Rationale: Transparent and complete reporting indicates a low risk of selective reporting bias.

Overall (Consensus): High risk

Rationale: Although most domains are rated as low risk, the presence of high risk in D2 (deviations from intended interventions) leads to an overall judgment of “high risk.” This suggests that performance-related bias may have influenced the study outcomes and should be considered when interpreting pooled estimates.

**10.ROB2 Assessment for Keleş**

D1 — Bias arising from the randomization process

R1 (Low risk): The study reports that participants were randomly assigned to intervention groups, indicating the use of an appropriate random sequence generation method.
R2 (Low risk): Although allocation concealment procedures are not described in detail, there is no indication of systematic imbalance between groups.
R3 (Low risk): Baseline characteristics appear comparable across groups, supporting the adequacy of the randomization process.

Consensus: Low risk
Rationale: Adequate randomization and balanced baseline characteristics suggest minimal risk of selection bias.

D2 — Bias due to deviations from the intended interventions

R1 (Some concerns): Participant blinding was not feasible, introducing moderate risk of bias.

R2 (High risk): Limited information on intervention fidelity suggests a higher likelihood of deviation from intended interventions.

R3 (Some concerns): While outcome assessment procedures may have been controlled, lack of participant blinding may still affect adherence and behavioral responses.

Consensus: Some concerns
Rationale: The behavioral nature of the intervention and lack of participant blinding introduce uncertainty regarding deviations from intended interventions.

D3 — Bias due to missing outcome data

R1 (Some concerns): The study reports outcome data, but detailed information regarding the extent and handling of missing data is limited.
R2 (Some concerns): There is no clear description of how missing data were addressed (e.g., intention-to-treat analysis or imputation methods).
R3 (Some concerns): It is unclear whether missing data may be related to outcomes, introducing potential attrition bias.

Consensus: Some concerns
Rationale: Limited reporting on missing data and handling methods introduces uncertainty regarding the potential impact of attrition on study results.

D4 — Bias in measurement of the outcome

R1 (Low risk): Outcomes were assessed using standardized and validated instruments.
R2 (Low risk): Measurement procedures appear to have been applied consistently across groups, reducing the likelihood of differential measurement bias.

R3 (Low risk): Outcome assessment methods appear appropriate and consistently implemented, and it is unlikely that measurement was influenced by knowledge of group allocation.

Consensus: Low risk
Rationale: The use of validated tools and standardized procedures minimizes bias in outcome assessment.

D5 — Bias in selection of the reported result

R1 (Low risk): Reported outcomes appear comprehensive and aligned with study objectives.

R2 (Some concerns): Lack of protocol registration or pre-specified analysis plan raises concerns about selective reporting.

R3 (Low risk): Although a pre-registered protocol or detailed analysis plan was not explicitly reported, the outcomes presented appear consistent with the study objectives, with no clear evidence of selective omission or reporting bias.

Consensus: Low risk
Rationale: Transparent and complete reporting indicates a low risk of selective reporting bias.

Overall (Consensus): Some concerns

Rationale: Although several domains are rated as low risk, the presence of some concerns in both D2 (deviations from intended interventions) and D3 (missing outcome data) leads to an overall judgment of “some concerns.” These limitations suggest cautious interpretation but do not substantially compromise the validity of the study findings.

**11.ROB2 Assessment for van Haaps et al.**

D1 — Bias arising from the randomization process

R1 (Some concerns): Randomization is mentioned but insufficiently described.

R2 (Low risk): No baseline imbalance is observed, suggesting acceptable randomization.

R3 (Some concerns): Although no major baseline imbalances are reported, insufficient detail limits confidence in the robustness of the randomization process.

Consensus: Some concerns
Rationale: Incomplete reporting of randomization and allocation concealment procedures introduces uncertainty regarding potential selection bias.

D2 — Bias due to deviations from the intended interventions

R1 (High risk): The intervention involved a virtual reality–based behavioral approach, and participant blinding was not feasible or not reported, which may substantially influence expectations and engagement.
R2 (High risk): There is insufficient information regarding adherence monitoring or whether deviations from intended interventions were controlled across groups.
R3 (High risk): The lack of participant blinding and limited reporting on intervention fidelity increase the likelihood that deviations from intended interventions may have influenced the outcomes.

Consensus: High risk
Rationale: The absence of participant blinding and insufficient control of intervention delivery introduce a substantial risk of performance bias.

D3 — Bias due to missing outcome data

R1 (High risk): A relatively high proportion of missing outcome data, with unclear handling methods, may bias the results.

R2 (Some concerns): Although missing data exist, there is no clear evidence that they are related to outcomes.

R3 (Some concerns): It remains unclear whether missing data may be related to outcomes, introducing potential attrition bias.

Consensus: Some concerns
Rationale: Limited reporting on missing data and handling strategies introduces uncertainty regarding potential bias.

D4 — Bias in measurement of the outcome

R1 (Low risk): Outcomes were assessed using standardized and validated measurement instruments.
R2 (Low risk): Measurement procedures appear consistent across groups, reducing the likelihood of differential measurement bias.

R3 (Low risk): Outcome assessment methods appear appropriate and consistently implemented, and it is unlikely that measurement was influenced by knowledge of group allocation.

Consensus: Low risk
Rationale: The use of validated tools and standardized assessment procedures minimizes measurement bias.

D5 — Bias in selection of the reported result

R1 (Low risk): The study reports outcomes comprehensively, including both significant and non-significant findings.
R2 (Low risk): There is no indication of selective outcome reporting.

R3 (Low risk): Reported outcomes appear complete and consistent with the study objectives, with no evidence of selective omission or preferential reporting.

Consensus: Low risk
Rationale: Transparent reporting suggests a low risk of selective reporting bias.

Overall (Consensus): High risk

Rationale: The presence of high risk in D2 (deviations from intended interventions), combined with additional concerns in D1 and D3, leads to an overall judgment of “high risk.” These limitations indicate that performance-related and methodological biases may have influenced the study outcomes and should be carefully considered when interpreting the pooled results.

**12.ROB2 Assessment for Kleiner et al.**

D1 — Bias arising from the randomization process

R1 (Low risk): The study reports that participants were randomly assigned to intervention groups, indicating the use of an appropriate random sequence generation method.
R2 (Low risk): Although allocation concealment procedures are not described in detail, there is no indication of systematic imbalance between groups.
R3 (Low risk): Baseline characteristics appear comparable across groups, supporting the adequacy of the randomization process.

Consensus: Low risk
Rationale: Adequate randomization and balanced baseline characteristics suggest minimal risk of selection bias.

D2 — Bias due to deviations from the intended interventions

R1 (Some concerns): The intervention involved a virtual reality–based behavioral approach, and participant blinding was not feasible, which may influence expectations and engagement.
R2 (Some concerns): Although the intervention protocol was likely standardized, the absence of participant blinding introduces potential performance bias.
R3 (Some concerns): While outcome assessment procedures may have been controlled, lack of participant blinding may still affect adherence and behavioral responses.

Consensus: Some concerns
Rationale: The behavioral nature of the intervention and lack of participant blinding introduce uncertainty regarding deviations from intended interventions.

D3 — Bias due to missing outcome data

R1 (Some concerns): Some missing outcome data are reported, but their impact on results is unclear.

R2 (Low risk): Missing data appear minimal and unlikely to affect conclusions.

R3 (Some concerns): It is unclear whether missing data may be related to outcomes, introducing potential attrition bias.

Consensus: Some concerns
Rationale: Limited reporting on missing data and handling methods introduces uncertainty regarding potential attrition bias.

D4 — Bias in measurement of the outcome

R1 (Low risk): Outcomes were assessed using validated instruments.

R2 (Some concerns): Lack of explicit assessor blinding introduces some uncertainty in measurement.

R3 (Low risk): Although assessor blinding was not explicitly reported, the use of validated instruments and consistent assessment procedures likely reduced the risk of measurement bias.

Consensus: Low risk
Rationale: The use of validated instruments and consistent procedures minimizes bias in outcome assessment.

D5 — Bias in selection of the reported result

R1 (Low risk): The study reports outcomes comprehensively, including both significant and non-significant findings.
R2 (Low risk): There is no indication of selective outcome reporting.

R3 (Low risk): Reported outcomes appear complete and aligned with the study objectives, with no evidence of selective omission or preferential reporting.

Consensus: Low risk
Rationale: Transparent and complete reporting indicates a low risk of selective reporting bias.

Overall (Consensus): Some concerns

Rationale: Although several domains are rated as low risk, the presence of some concerns in both D2 (deviations from intended interventions) and D3 (missing outcome data) leads to an overall judgment of “some concerns.” These limitations suggest cautious interpretation but do not substantially compromise the validity of the study findings.
