# Supplementary material for: Effects of virtual reality interventions on anxiety symptoms in women undergoing gynecological examinations and surgeries: a multi-level dose–response meta-analysis
Source: Front Psychol. 2026 May 29;17:1792559. doi: 10.3389/fpsyg.2026.1792559 (PMC13260798; doi:10.3389/fpsyg.2026.1792559)
Supplement: Supplementary file 2 [file Table_2.docx]

**Table of Contents**

[Supplementary document Retrieval Strategy 1](#_Toc24072)

[Figure 1.Standardized residuals and Cook’s distance threshold plot at the intervention endpoint 6](#_Toc10029)

[Figure 2.Sensitivity analysis plot at the intervention endpoint 6](#_Toc24718)

[Figure 3.Trim-and-fill plot at the intervention endpoint 7](#_Toc29869)

[Figure 4.Multilevel variance decomposition and I² contributions 8](#_Toc15230)

[Table 1.Inter-Rater Agreement Results for Each ROB2 Domain 9](#_Toc20910)

[Study selection consistency and Cohen’s κ 9](#_Toc12967)

[Table 2.Summary of Findings and GRADE Assessment for Anxiety Outcomes 11](#_Toc15054)

[Figure 5.Trial Sequential Analysis (TSA) 12](#_Toc8208)

[Figure 6.Interval plot 13](#_Toc14933)

[Figure 7.ECDF comparison 14](#_Toc981)

[Figure 8.Optimal Dose Posterior Distribution Plot 15](#_Toc15127)

[Table 3.Results of Leave-One-Out Sensitivity Analysis (Top 5 Most Influential Studies) 16](#_Toc1022)

[Figure 9.Forest plot of multilevel meta-analysis 17](#_Toc8056)

[Table 4. Subgroup analysis of the effects of virtual reality interventions on anxiety outcomes in women undergoing gynecological examinations and surgical procedures. 18](#_Toc25579)

# Supplementary document Retrieval Strategy

**1. PubMed**

("Virtual Reality"[Mesh] OR "virtual reality"[tiab] OR VR[tiab] OR "immersive virtual environment*"[tiab])

AND

("Gynecologic Examination"[Mesh]

OR "Gynecologic Surgical Procedures"[Mesh]

OR gynecolog*[tiab]

OR gynaecolog*[tiab]

OR "pelvic examination"[tiab]

OR "gynecologic examination"[tiab]

OR "gynecologic surgery"[tiab]

OR "gynecological procedure*"[tiab])

AND

("Anxiety"[Mesh]

OR "Anxiety Disorders"[Mesh]

OR "Stress, Psychological"[Mesh]

OR anxiety[tiab]

OR anxious[tiab]

OR stress[tiab]

OR distress[tiab]

OR fear[tiab]

OR "State-Trait Anxiety Inventory"[tiab]

OR STAI[tiab])

AND

("Systematic Review"[pt]

OR "Meta-Analysis"[pt]

OR "systematic review"[tiab]

OR "meta-analysis"[tiab])

Filters: English, Publication date to 2026/1/05

**2. Cochrane Library**

#1 [mh "Virtual Reality"]

OR "virtual reality":ti,ab,kw

OR VR:ti,ab,kw

#2 [mh "Gynecologic Examination"]

OR [mh "Gynecologic Surgical Procedures"]

OR gynecolog*:ti,ab,kw

OR gynaecolog*:ti,ab,kw

OR "pelvic examination":ti,ab,kw

#3 [mh Anxiety]

OR [mh "Anxiety Disorders"]

OR [mh "Psychological Stress"]

OR anxiety:ti,ab,kw

OR stress:ti,ab,kw

OR distress:ti,ab,kw

OR fear:ti,ab,kw

OR STAI:ti,ab,kw

#4 [mh "Systematic Reviews as Topic"]

OR "systematic review":ti,ab,kw

OR "meta-analysis":ti,ab,kw

#5 #1 AND #2 AND #3 AND #4

Publication Date to January 5, 2026, Language: English

**3. Web of Science**

TS=("virtual reality"

OR VR

OR "immersive virtual environment*")

AND

TS=(gynecolog*

OR gynaecolog*

OR "pelvic examination"

OR "gynecologic examination"

OR "gynecologic surgery"

OR "gynecological procedure*")

AND

TS=(anxiety

OR anxious

OR stress

OR distress

OR fear

OR STAI

OR "state trait anxiety")

AND

TS=("systematic review"

OR "meta-analysis")

Timespan: All years to January 5, 2026

**4. PsycINFO**

1. exp Virtual Reality/

OR ("virtual reality" OR VR).ti,ab.

2. exp Gynecology/

OR exp Gynecologic Surgery/

OR (gynecolog* OR gynaecolog*

OR "pelvic examination").ti,ab.

3. exp Anxiety/

OR exp Anxiety Disorders/

OR exp Psychological Stress/

OR (anxiety OR stress OR distress

OR fear OR STAI).ti,ab.

4. exp Systematic Review/

OR exp Meta Analysis/

OR ("systematic review"

OR "meta-analysis").ti,ab.

5. 1 AND 2 AND 3 AND 4

6. limit 5 to (english language and journal article and human)


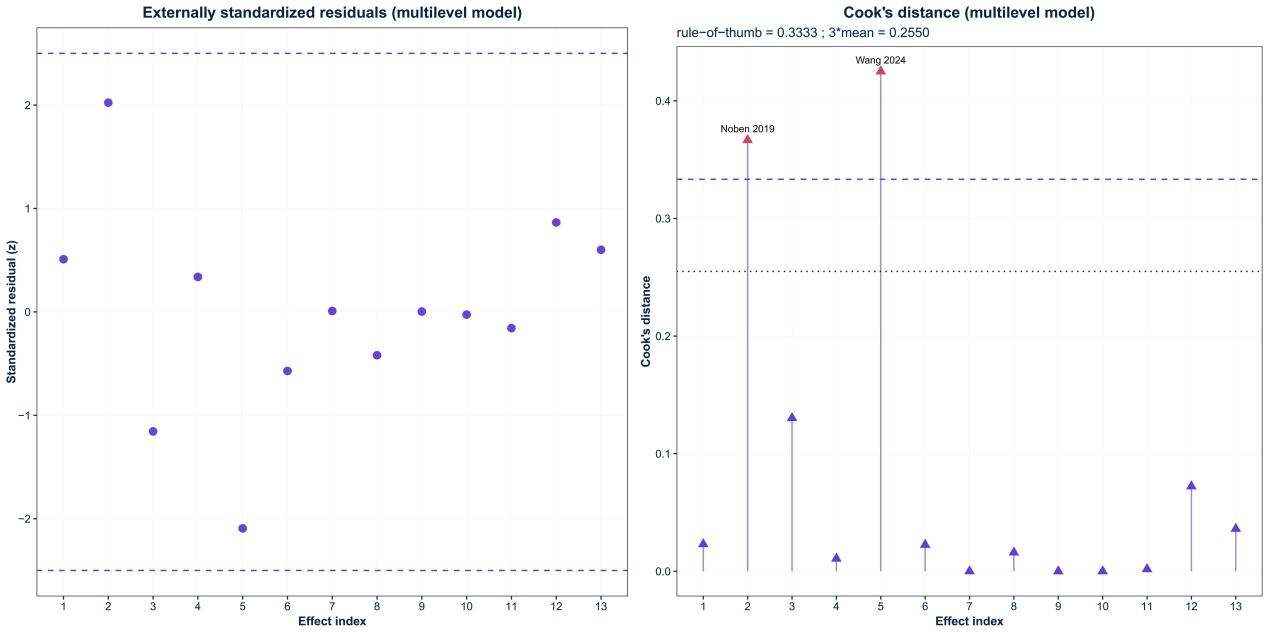


# **Figure 1.**Standardized residuals and Cook’s distance threshold plot at the intervention endpoint


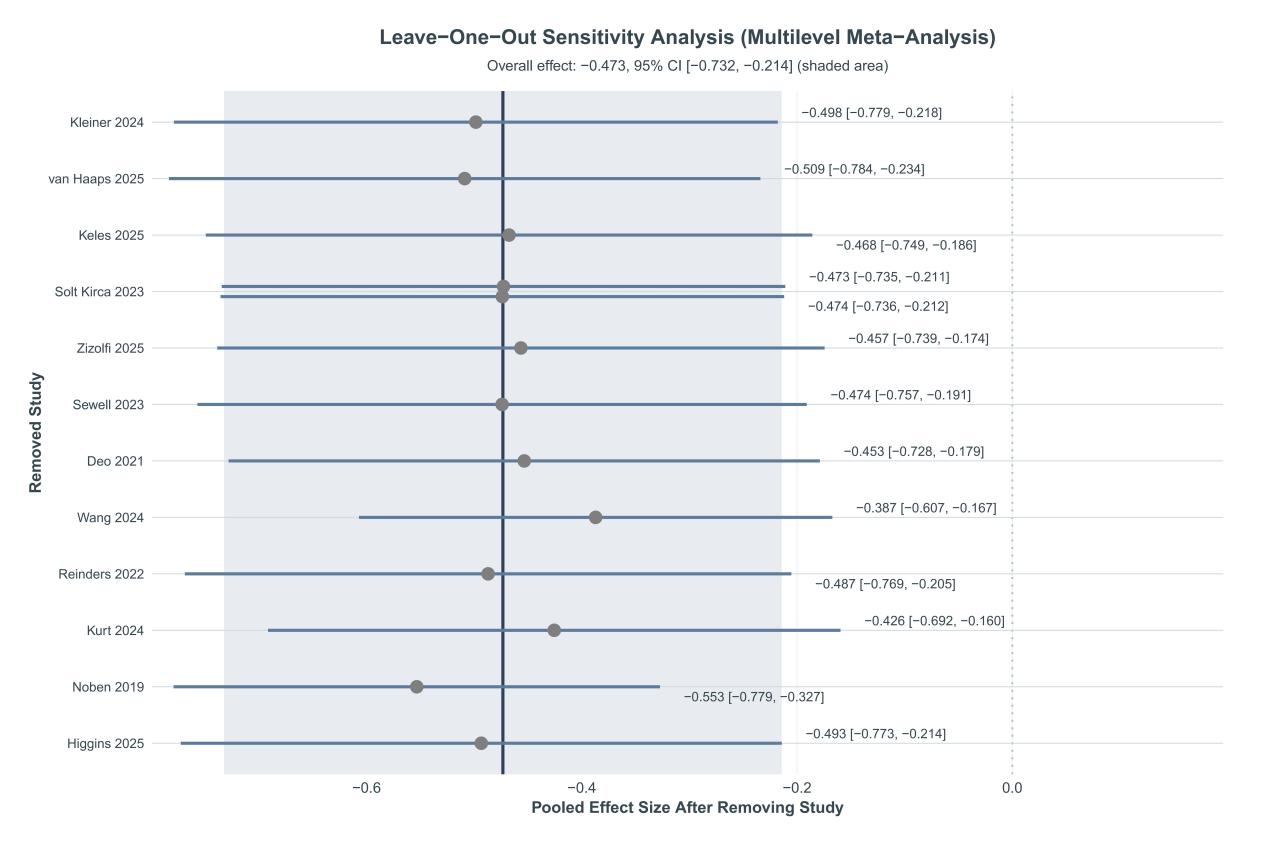


# **Figure 2.**Sensitivity analysis plot at the intervention endpoint


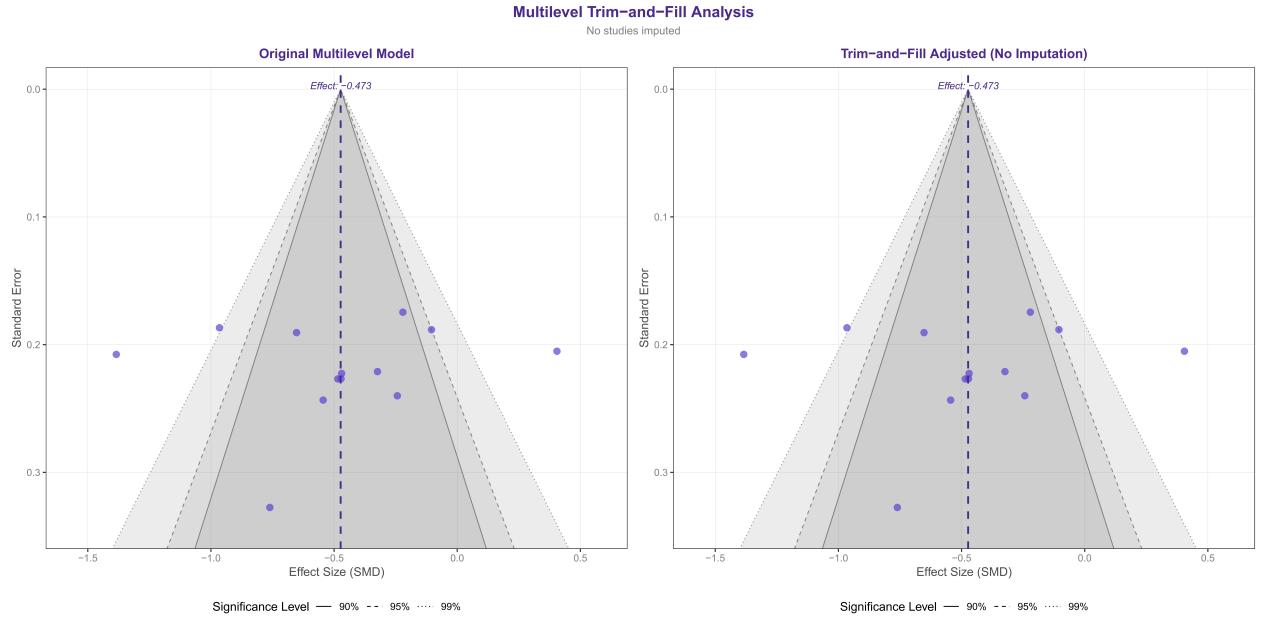


# **Figure 3.**Trim-and-fill plot at the intervention endpoint


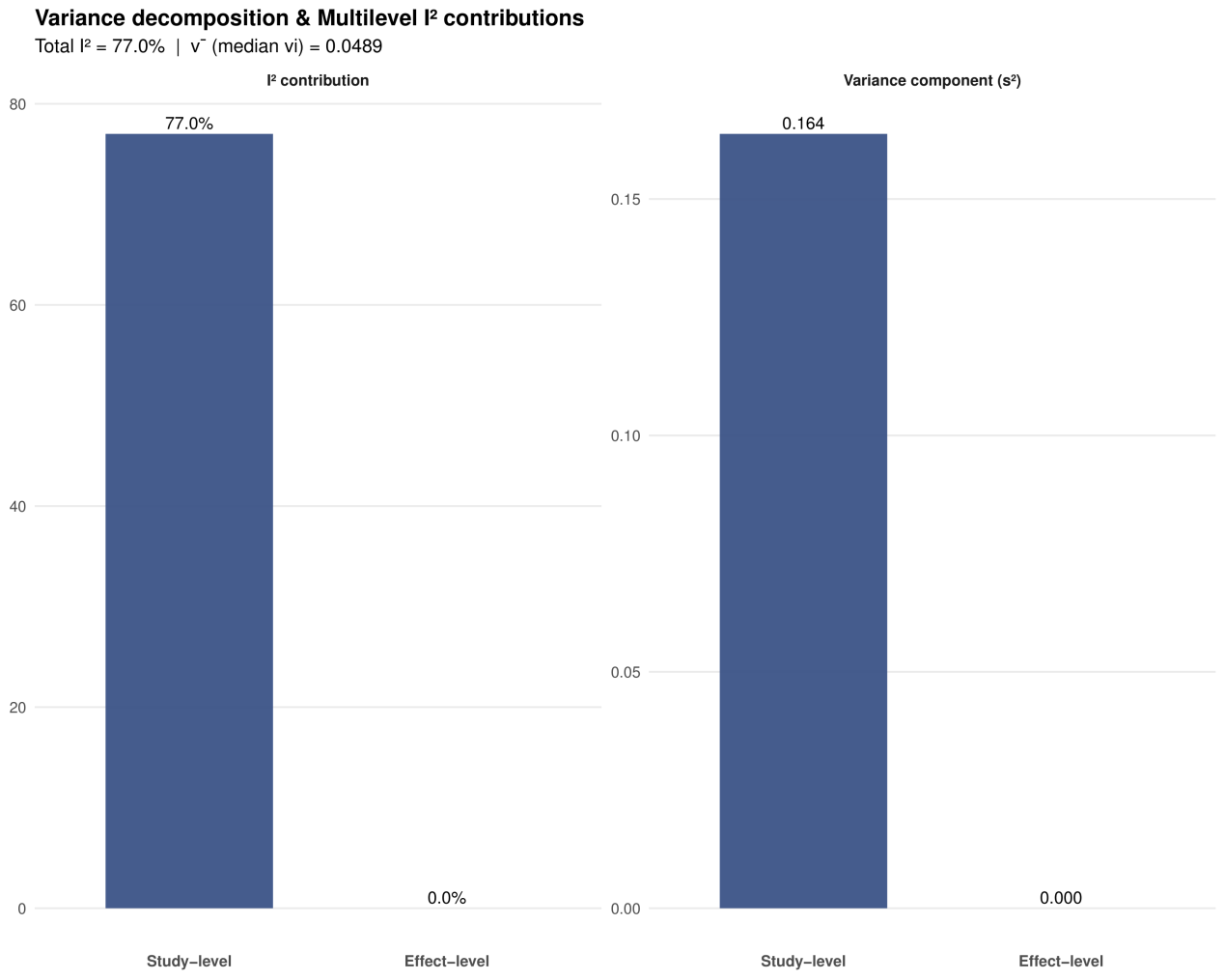


# **Figure 4.**Multilevel variance decomposition and I² contributions

# **Table 1.**Inter-Rater Agreement Results for Each ROB2 Domain

The table below displays the simple agreement rate, Cohen's Kappa, and Weighted Kappa calculated separately for each domain (D1-D5).

| **Domain** | **Simple Agreement Rate** | **Cohen’s Kappa** | **Weighted Kappa** | **Strength of Agreement (Cohen’s Kappa)** |
| --- | --- | --- | --- | --- |
| D1 (Randomization process) | 83.3% (10/12) | -0.091 | -0.091 | Poor |
| D2 (Deviations from intended interventions) | 58.3% (7/12) | 0.310 | 0.412 | Fair |
| D3 (Missing outcome data) | 58.3% (7/12) | 0.341 | 0.474 | Fair |
| D4 (Measurement of the outcome) | 83.3% (10/12) | -0.091 | -0.091 | Poor |
| D5 (Selection of the reported result) | 91.7% (11/12) | 0.000 | 0.000 | Slight |

**Note:** The inter-rater agreement between the two reviewers for each ROB2 domain was assessed. The Simple Agreement Rate​ was calculated as the percentage of studies for which both reviewers assigned identical judgments (Low, Some concerns, or High). Cohen's Kappa (κ)​ statistic, which accounts for chance agreement, was calculated from 3x3 contingency tables. Weighted Kappa​ was also computed using linear weights (0, 0.5, 1) to grant partial credit for adjacent rating disagreements (e.g., Low vs. Some concerns). The strength of agreement based on Cohen's Kappa was interpreted as follows: 0.81–1.00 = "Perfect/Almost Perfect", 0.61–0.80 = "Substantial/Good", 0.41–0.60 = "Moderate", 0.21–0.40 = "Fair", 0.00–0.20 = "Slight", and <0.00 = "Poor".

# Study selection consistency and Cohen’s κ

**1. Title and abstract screening (updated)**

Based on a minimally adjusted 2×2 contingency table—constructed to ensure that a+b+c=276and that the total number of records screened was 592—both reviewers included 204 records, J.L. included while H.R. excluded 36 records, J.L. excluded while H.R. included 36 records, and both reviewers excluded 316 records (total n=592). According to the PRISMA flow diagram, 592 records were screened at the title and abstract stage, of which 276 were retained for retrieval and 316 were excluded (including 227 records unrelated to Traditional Chinese Manipulative Bone-setting Therapy (TCMBP) and 89 individual case reports). The observed agreement was Po​=na+d​=592204+316​≈0.8784, while the expected agreement was Pe​≈0.5179. Therefore, Cohen’s κ coefficient was calculated as κ=1−Pe​Po​−Pe​​≈0.7477. The approximate standard error of κ was 0.0279, yielding a 95% confidence interval (CI) of 0.693–0.802 (reported as 0.69–0.80 after rounding). The corresponding z statistic was 26.83 (p<0.001). According to the criteria proposed by Landis and Koch, a κ value of approximately 0.75 indicated substantial agreement​ between the two reviewers.

#### **2. Full-Text Eligibility Assessment (Updated)**

Based on a minimally adjusted 2×2 contingency table—constructed to ensure that a+b+c=180and that the total number of reports screened was 206—both reviewers included 180 reports, J.L. included while H.R. excluded 10 reports, J.L. excluded while H.R. included 10 reports, and both reviewers excluded 16 reports (total n=206). According to the PRISMA flow diagram, 206 reports were sought for retrieval during the full-text eligibility assessment stage, of which 180 were assessed for eligibility, 26 were not retrieved, 180 were assessed as eligible, and 168 were excluded for the following reasons: Irrelevant intervention (68), Irrelevant comparator (33), No control group (40), and Irrelevant Outcome Indicators (27). The observed agreement was P_o = \fra ca+dn=206180+16​≈0.9515, while the expected agreement was Pe​≈05000. Therefore, Cohen.’s κ coefficient was calculated as κ=1−Pe​Po​−Pe​​≈0.9030. The approximate standard error of κ was 0.0215, yielding a 95% confidence interval (CI) of 0.861–0.945 (reported as 0.86–0.95 after rounding). The corresponding z statistic was 42.00 (p<0.001). According to the criteria proposed by Landis and Koch, a κ value of approximately 0.90 indicated almost perfect agreement​ between the two reviewers.

**3. Short version**

Based on the provided statistical report, here is a concise summary of the inter-rater agreement for the study selection process:Inter-rater agreement between the two reviewers was substantial at the title and abstract screening stage (κ = 0.75, 95% CI 0.69–0.80) and almost perfect at the full-text screening stage (κ = 0.90, 95% CI 0.86–0.95), indicating high consistency throughout the study selection process.

# **Table 2.**Summary of Findings and GRADE Assessment for Anxiety Outcomes

| **GRADE domain** | **Judgment** | **Rationale** |
| --- | --- | --- |
| Risk of bias | Serious (downgraded 1 level) | Although all included studies were randomized controlled trials, the overall RoB 2 assessment indicated “some concerns” across most studies. Issues were primarily observed in domains D1 (randomization process), D2 (deviations from intended interventions, particularly due to lack of blinding), D3 (missing outcome data), and D5 (selection of reported results). Domain D4 (measurement of the outcome) was consistently rated as low risk. Therefore, the evidence was downgraded by one level due to risk of bias. |
| Inconsistency | Serious (downgraded 1 level) | Substantial heterogeneity was observed across studies (I² = 77%, p < 0.001), indicating considerable between-study variability. Although subgroup analyses and meta-regression partially explained heterogeneity, residual inconsistency remained, justifying downgrading for inconsistency. |
| Indirectness | Not downgraded | The population (women undergoing gynecological examinations and surgeries), intervention (virtual reality), comparators (routine care or standard interventions), and outcome (validated anxiety scales such as STAI-S, HADS-A, VAS, and NRS) were directly aligned with the research question. |
| Imprecision | Not downgraded | The pooled effect size was statistically significant (SMD = −0.47, 95% CI −0.73 to −0.21) and did not cross the null value. Although the confidence interval was moderately wide, it consistently indicated a beneficial effect, and TSA confirmed sufficient cumulative evidence. |
| Publication bias | Not downgraded | Egger’s regression test showed no significant publication bias (p = 0.881). Trim-and-fill analysis indicated no missing studies, and sensitivity analyses confirmed the robustness of the results. |
| Overall quality of evidence | **Moderate** | The certainty of evidence was initially rated as high because all included studies were RCTs. It was downgraded by one level due to risk of bias and one level due to inconsistency; however, given the robustness of the findings and supporting analyses (sensitivity analysis, TSA), the overall quality of evidence was judged as moderate. |


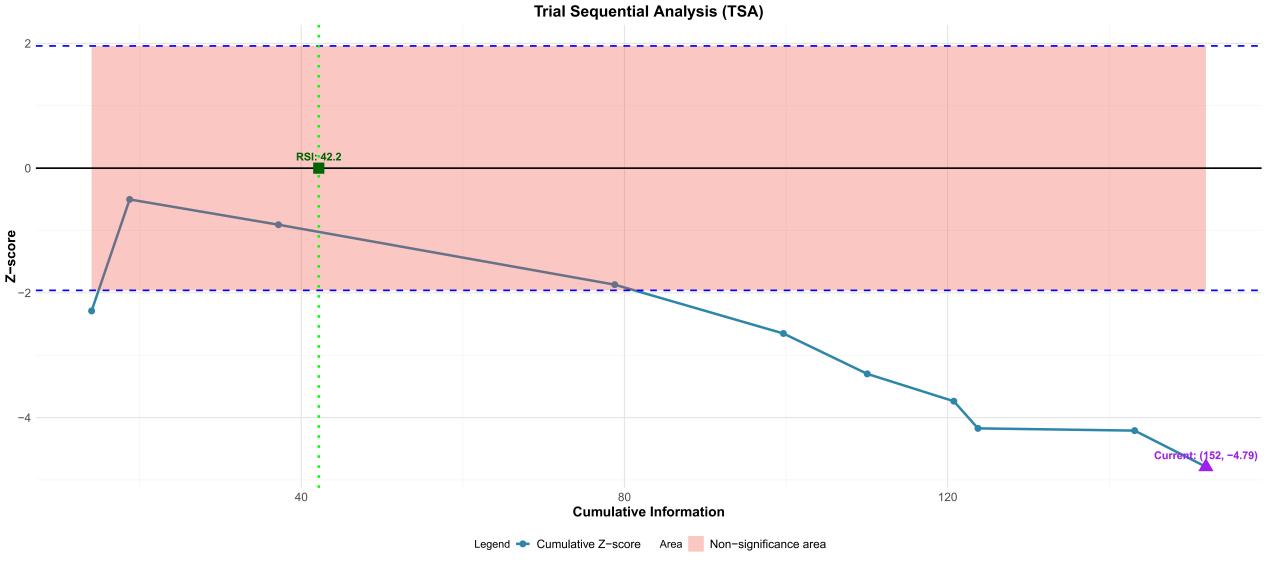


# **Figure 5.**Trial Sequential Analysis (TSA)


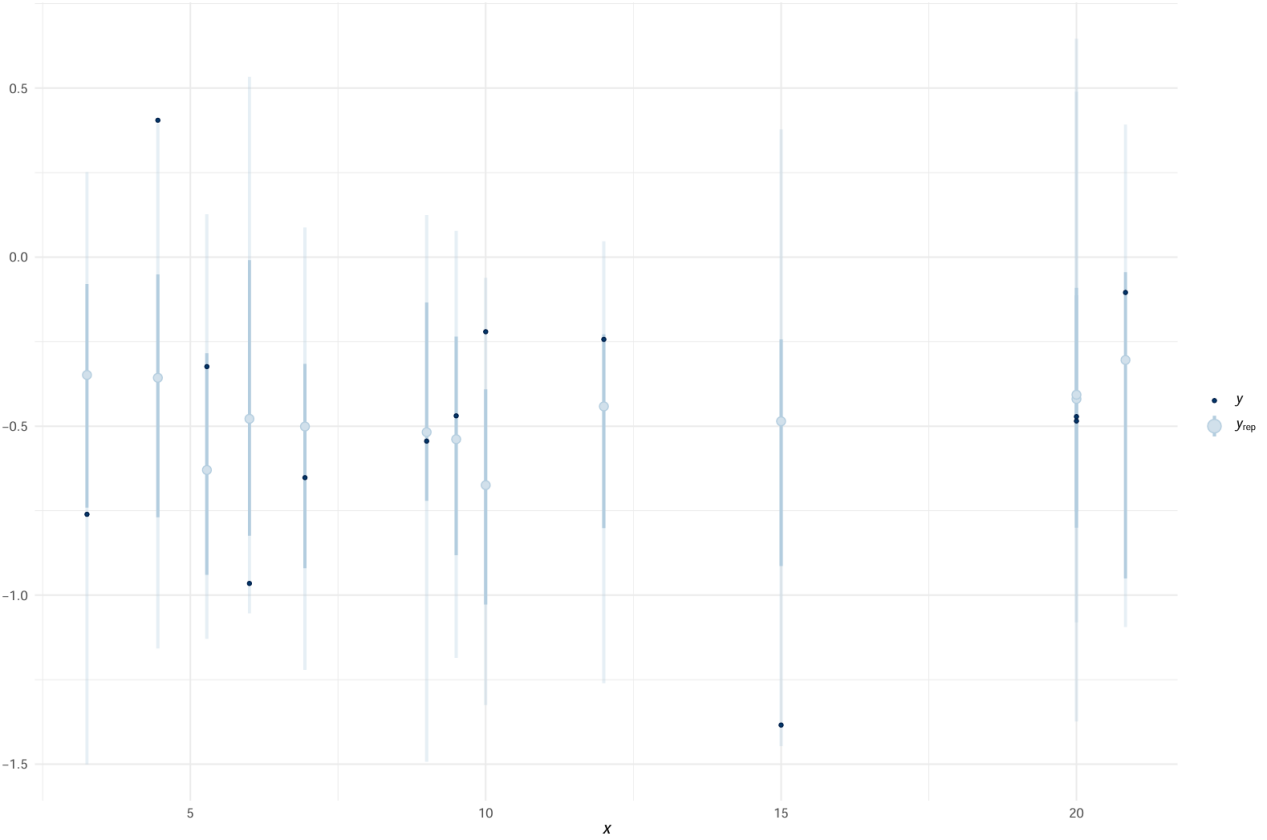


# **Figure 6.**Interval plot


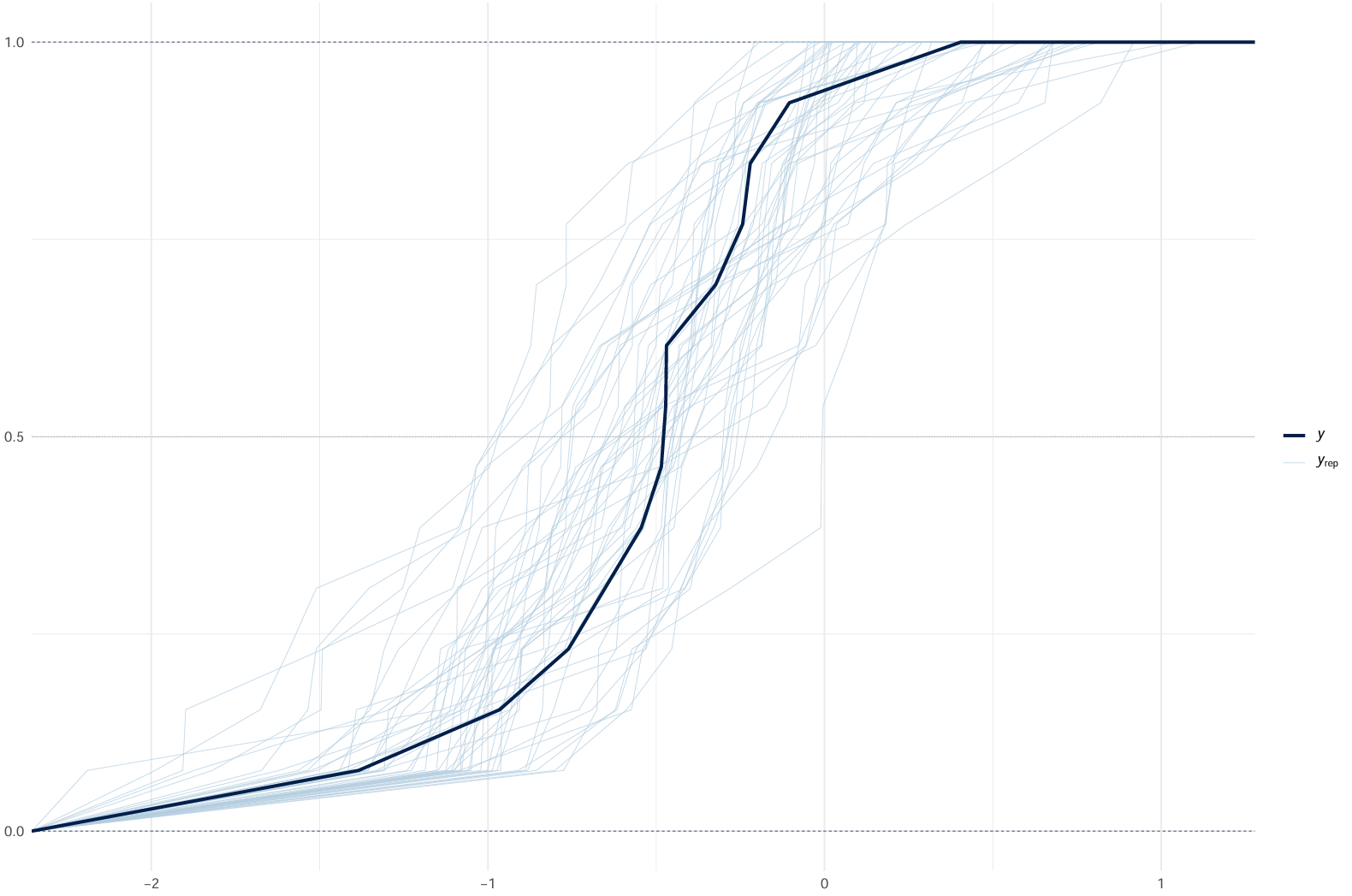


# **Figure 7.**ECDF comparison


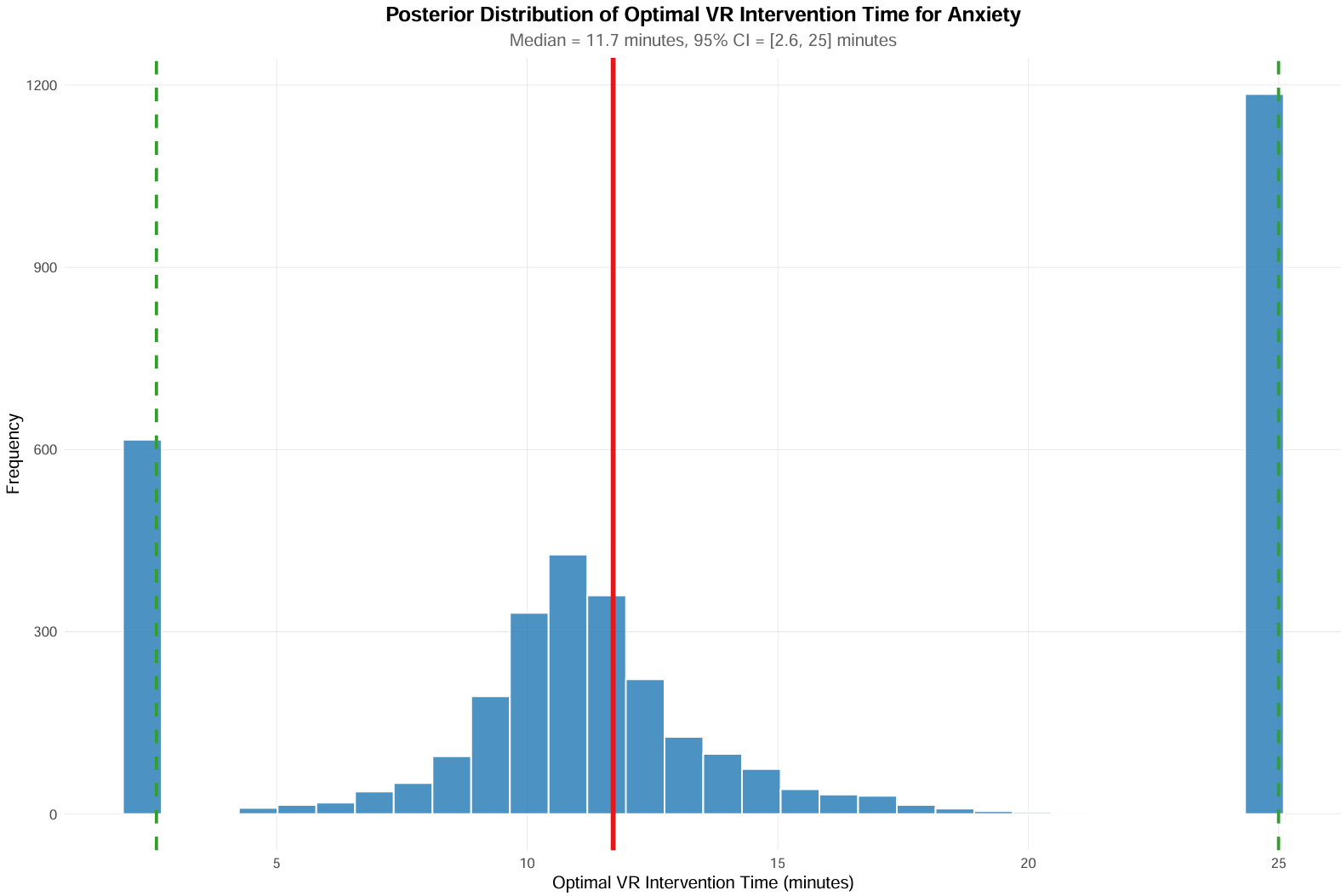


# **Figure 8.**Optimal Dose Posterior Distribution Plot

# **Table 3.**Results of Leave-One-Out Sensitivity Analysis (Top 5 Most Influential Studies)

| Study | Effect after removal | Deviation | Significant deviation |
| --- | --- | --- | --- |
| Wang 2024 | -0.387 [-0.607, -0.167] | +0.086 | No |
| Noben 2019 | -0.553 [-0.779, -0.327] | -0.080 | No |
| Kurt 2024 | -0.426 [-0.692, -0.160] | +0.048 | No |
| van Haaps 2025 | -0.509 [-0.784, -0.234] | -0.036 | No |
| Kleiner 2024 | -0.498 [-0.779, -0.218] | -0.025 | No |


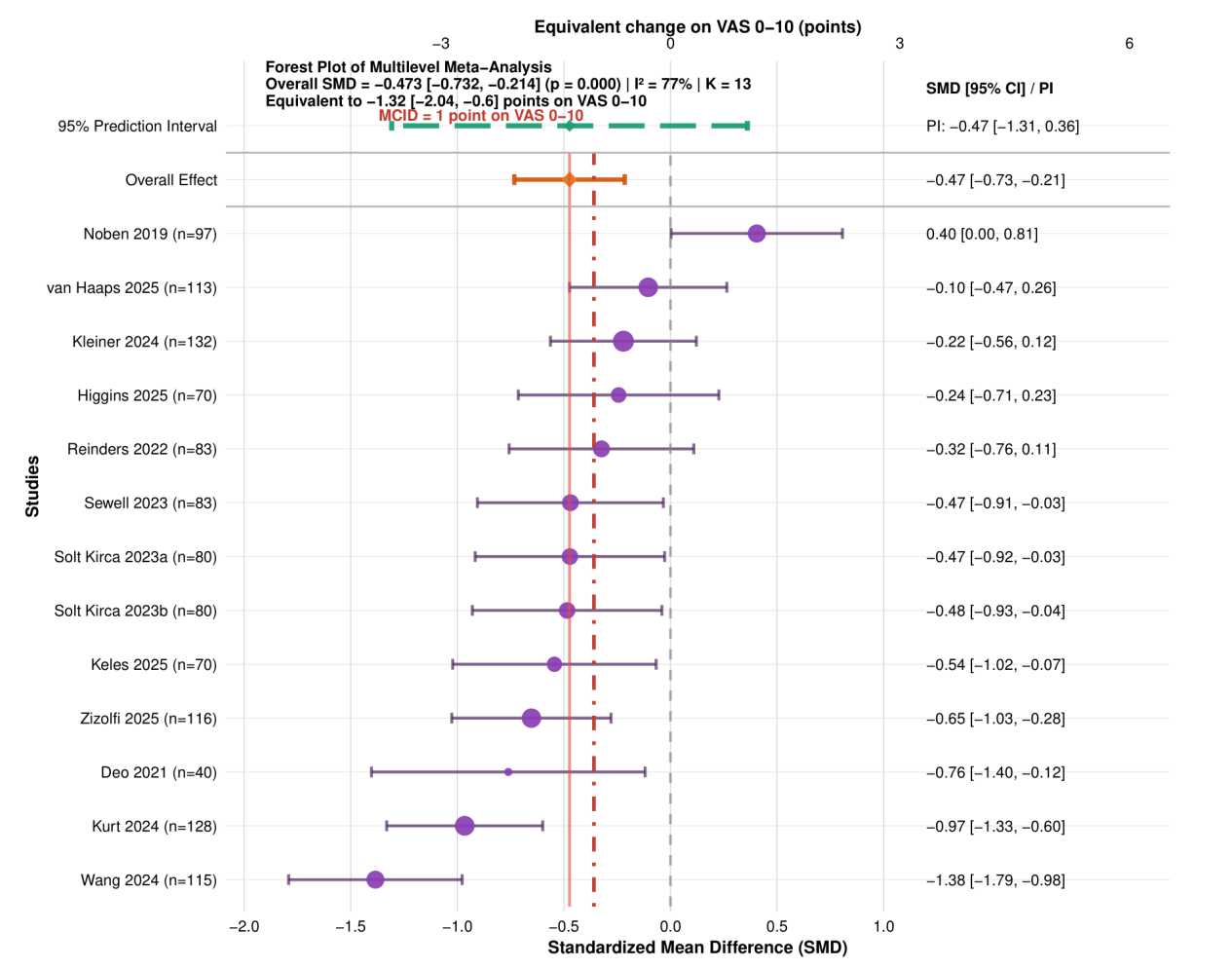


# **Figure 9.**Forest plot of multilevel meta-analysis

# Table 4. Subgroup analysis of the effects of virtual reality interventions on anxiety outcomes in women undergoing gynecological examinations and surgical procedures.

| Dimensionality | sort | K | N | I^2^ | Effect model | SMD and 95%CI | GRADE | P | P-interaction​​​ |
| --- | --- | --- | --- | --- | --- | --- | --- | --- | --- |
| Nation |  |  |  |  |  |  |  |  | 0.001* |
|  | Australia​ | 1 | 70 | * | Random | -0.24(-0.71, 0.23) | Very Low | 0.311 |  |
|  | China | 1 | 115 | * | Random | -1.38(-1.79, -0.98) | Low | 0.001* |  |
|  | Israel | 1 | 132 | * | Random | -0.22(-0.56, 0.12) | Very Low | 0.206 |  |
|  | Italy | 1 | 116 | * | Random | -0.65(-1.03, -0.28) | Low | 0.001* |  |
|  | The Netherlands | 3 | 293 | 68.5% | Random | -0.004(-0.42, 0.41) | Very Low | 0.983 |  |
|  | Turkey | 4 | 508 | 28.1% | Random | -0.64(-0.90, -0.38) | Moderate | 0.001* |  |
|  | UK​ | 2 | 123 | 0% | Random | -0.56(-0.92, -0.20) | Moderate | 0.002* |  |
| Subject_type |  |  |  |  |  |  |  |  | 0.171 |
|  | Highly invasive | 4 | 452 | 92% | Random | -0.48(-1.21, 0.24) | Very Low | 0.189 |  |
|  | Minimally invasive | 7 | 637 | 12% | Random | -0.36(-0.53, -0.19) | Moderate | 0.001* |  |
|  | Non-invasive | 2 | 268 | 46.7% | Random | -0.78(-1.19, -0.38) | Moderate | 0.001* |  |
| Intervention mode1 |  |  |  |  |  |  |  |  | 0.083 |
|  | VR | 8 | 865 | 72.6% | Random | -0.64(-0.92, -0.35) | Moderate | 0.001* |  |
|  | VR＋ | 5 | 492 | 75.4% | Random | -0.23(-0.59, 0.13) | Very Low | 0.214 |  |
|  |  |  |  |  |  |  |  |  |  |
| Intervention  time |  |  |  |  |  |  |  |  | 0.575 |
|  | ＜5 | 2 | 137 | 89% | Random | -0.15(-1.29, 0.99) | Very Low | 0.797 |  |
|  | 5-10 | 6 | 682 | 49.5% | Random | -0.53(-0.76, -0.30) | Very Low | 0.001* |  |
|  | 11-15 | 2 | 185 | 92.2% | Random | -0.82(-1.94, 0.30) | Low | 0.151 |  |
|  | 16-20 | 3 | 353 | 12.5% | Random | -0.33(-0.59, -0.06) | Low | 0.016 |  |
| Scale type |  |  |  |  |  |  |  |  | 0.001* |
|  | HADS-A | 1 | 115 | * | Random | -1.38(-1.79, -0.98) | Low | 0.001* |  |
|  | NRS | 3 | 239 | 0% | Random | -0.61(-0.87, -0.35) | Moderate | 0.001* |  |
|  | STAI-S | 7 | 836 | 54.4% | Random | -0.44(-0.67, -0.22) | Moderate | 0.001* |  |
|  | VAS | 2 | 167 | 76.3% | Random | 0.09(-0.54, 0.73) | Very Low | 0.774 |  |
| ROB2_Domain |  |  |  |  |  |  |  |  | 0.001* |
|  | High | 5 | 476 | 0.1% | Random | -0.40(-0.61, -0.19) | Low | 0.001* |  |
|  | Low | 1 | 115 | * | Random | -1.38(-1.79,-0.98) | Low | 0.001* |  |
|  | Some concerns | 7 | 766 | 78.7% | Random | -0.37(-0.69,-0.04) | Low | 0.027* |  |
| Intervention mode2 |  |  |  |  |  |  |  |  | 0.2657 |
|  | Conscious Sedation Control | 1 | 113 | * | Random | -0.80(-1.72,0.12) | Very Low | 0.088 |  |
|  | Procedural Information Control | 1 | 97 | * | Random | -0.81(-1.73,0.11) | Very Low | 0.085 |  |
|  | Skin-to-Skin Care Control | 1 | 120 | * | Random | -0.56(-1.25,0.13) | Very Low | 0.109 |  |
|  | Standard Care | 9 | 944 | 6.1% | Random | -0.06(-0.40,0.27) | Very Low | 0.665 |  |
|  | Standardized Procedural Information Control | 1 | 83 | * | Random | 0.18(-0.70,1.05) | Very Low | 0.695 |  |
